# Assessing age, breeding stage, and mating activity as drivers of variation in the reproductive microbiome of female tree swallows

**DOI:** 10.1002/ece3.7929

**Published:** 2021-07-29

**Authors:** Jessica Hernandez, Catherine Hucul, Emily Reasor, Taryn Smith, Joel W. McGlothlin, David C. Haak, Lisa K. Belden, Ignacio T. Moore

**Affiliations:** ^1^ Department of Biological Sciences Virginia Tech Blacksburg VA USA; ^2^ School of Plant and Environmental Sciences Virginia Tech Blacksburg VA USA

**Keywords:** extra‐pair paternity, hormone implant, mating strategy, reproductive microbiome, tree swallows

## Abstract

Sexually transmitted microbes are hypothesized to influence the evolution of reproductive strategies. Though frequently discussed in this context, our understanding of the reproductive microbiome is quite nascent. Indeed, testing this hypothesis first requires establishing a baseline understanding of the temporal dynamics of the reproductive microbiome and of how individual variation in reproductive behavior and age influence the assembly and maintenance of the reproductive microbiome as a whole. Here, we ask how mating activity, breeding stage, and age influence the reproductive microbiome. We use observational and experimental approaches to explain variation in the cloacal microbiome of free‐living, female tree swallows (*Tachycineta bicolor*). Using microsatellite‐based parentage analyses, we determined the number of sires per brood (a proxy for female mating activity). We experimentally increased female sexual activity by administering exogenous 17ß‐estradiol. Lastly, we used bacterial 16S rRNA amplicon sequencing to characterize the cloacal microbiome. Neither the number of sires per brood nor the increased sexual activity of females significantly influenced female cloacal microbiome richness or community structure. Female age, however, was positively correlated with cloacal microbiome richness and influenced overall community structure. A hypothesis to explain these patterns is that the effect of sexual activity and the number of mates on variation in the cloacal microbiome manifests over an individual's lifetime. Additionally, we found that cloacal microbiome alpha diversity (Shannon Index, Faith's phylogenetic distance) decreased and community structure shifted between breeding stages. This is one of few studies to document within‐individual changes and age‐related differences in the cloacal microbiome across successive breeding stages. More broadly, our results contribute to our understanding of the role that host life history and behavior play in shaping the cloacal microbiomes of wild birds.

## Graphical abstract

In this paper, we ask the question: how do mating activity, breeding stage, and age influence female reproductive microbiomes? We address this question using observational and experimental approaches to explain both between‐ and within‐individual variation in the cloacal microbiome of free‐living, female birds that exhibit differences in extra‐pair paternity rates. We found that female age and breeding stage, but not number of mates or experimentally elevated sexual activity (via 17ß‐estradiol implants), significantly influenced cloacal microbiome alpha diversity and explained variation in bacterial community structure.

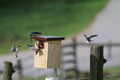

## 1 INTRODUCTION

The social and reproductive behavior of animals can shape the diverse microbial communities (“microbiomes”) that live in and on their bodies (Archie & Tung, 2015; Dugatkin, 2020; Ezenwa et al., 2012; Münger et al., 2018; Westneat, 1987). Animal mating behaviors, for instance, can transmit microbes that become incorporated into the host‐associated reproductive microbiome. Considerable theoretical work has explored the potential transmission of microbes through mating (Boots & Knell, 2002; Graves & Duvall, 1995; Hamilton, 1990; Kokko et al., 2002; Lockhart et al., 1996; Loehle, 1995; Lombardo, 1998; Lombardo et al., 1999; Poiani, 2010; Sheldon, 1993; Thrall et al., 1997, 2000), but relatively few empirical studies focusing on the reproductive microbiome of wild animals exist. Further, though this theoretical work has advanced our understanding of the transmission and potential effects of pathogenic microbes, it has largely ignored the reproductive microbiome as a whole. Microbes in the reproductive tract have the potential to considerably impact host reproductive function and thus may play a significant role in shaping the evolution of mate choice, sexual conflict, reproductive isolation, and mating systems more broadly (reviewed in Rowe et al., 2020).

In birds, amphibians, reptiles, cartilaginous fishes (i.e., chondrichthyans), and some mammals (i.e., monotremes), the cloaca is the reproductive structure of both sexes. The cloaca harbors a diverse community of microbes (hereafter the “cloacal microbiome”) as the terminus for the digestive and reproductive tracts and the site of contact and ejaculate transfer during copulation (e.g., Kulkarni & Heeb, 2007; Westneat & Rambo, 2000; White et al., 2011). The cloacal microbiome consists of a combination of beneficial, commensal, and pathogenic microbes and can be shaped by a variety of factors, including host life history, environment, and behavior (Rowe et al., 2020). Within‐individual changes in the phylogenetic diversity of male cloacal bacteria between breeding and nonbreeding stages suggest that physiology and overall breeding condition may also play a role in shaping the cloacal microbiome (Escallón et al., 2019).

Socially monogamous species that engage in extra‐pair copulations exhibit individual differences in the number of mates that they have during the breeding season and thus likely vary in their exposure to sexually transmitted microbes. While evidence of extra‐pair copulations in socially monogamous species has been documented in insects (e.g., Dillard, 2017), fish (e.g., Avise et al., 2002), amphibians (e.g., Liebgold et al., 2006), reptiles (e.g., Uller & Olsson, 2008), and mammals (e.g., Cohas & Allainé, 2009), birds are arguably the most well studied (e.g., Griffith et al., 2002) and the focus of this study. In birds, most studies examining the impact of mating behavior on reproductive microbiomes have selectively targeted a limited set of bacteria in social partners (Hupton et al., 2003; Lombardo & Thorpe, 2000; Stewart & Rambo, 2000) or have focused on interspecific comparisons of species with divergent mating systems using culture‐dependent approaches (e.g., monogamous vs. polygamous: Poiani & Gwozdz, 2002; Poiani & Wilks, 2000a, 2000b). Culture‐independent work exploring how the reproductive microbiome is shaped with respect to mating has focused on genetically monogamous systems and/or pair‐bonded social partners (Ambrosini et al., 2019; Hernandez et al., 2020; Kreisinger et al., 2015; White et al., 2010). For instance, pairs of genetically monogamous black‐legged kittiwakes (*Rissa tridactyla*) exhibit similar cloacal bacterial communities when allowed to copulate, and this similarity was reduced when pairs were experimentally blocked from mating (White et al., 2010). Moreover, in barn swallows (*Hirundo rustica*), a socially monogamous species that exhibits extra‐pair mating activity, social partners exhibited more similar cloacal bacterial communities than expected by chance, though one study acknowledged a low effect size with regard to this pattern (Ambrosini et al., 2019; Kreisinger et al., 2015). In contrast, the cloacal bacterial communities of tree swallow (*Tachycineta bicolor*) social partners were not more similar than expected by chance (Hernandez et al., 2020). However, these studies have not focused on variation in sexual activity and the number of sexual partners, two factors that are hypothesized to contribute to variation in cloacal microbiome diversity.

Here, we test the hypothesis that mating activity influences reproductive microbiome richness and community structure using both an observational and an experimental approach. First, we compared natural variation in the number of sires represented in a brood (a proxy for female mating activity) and the diversity of the cloacal microbiome of females in a population of tree swallows. We predicted that females with more sires within their brood would have increased cloacal bacterial richness and that the number of sires would explain variation in the structure of the bacterial community. Similarly, we predicted that older females would have higher cloacal bacterial richness compared to younger females, presumably in part due to the increased exposure to bacteria from previous mating opportunities that have occurred over their lifetime. Second, we performed an experimental study administering exogenous 17*ß*‐estradiol (hereafter, “estradiol”) using silastic implants to increase female sexual activity and compared cloacal microbiome diversity to control females given blank implants. Exogenously administered estradiol has been consistently found to be positively associated with increased solicitations and copulations in female birds (e.g., Leboucher et al., 1998; Moore, 1982). We predicted that females implanted with exogenous estradiol would more actively solicit copulations, both from their social mate and from other males, and would thus have higher cloacal microbiome richness compared to control females.

## 2 METHODS

### 2.1 Study system and study site

**FIGURE 1:**
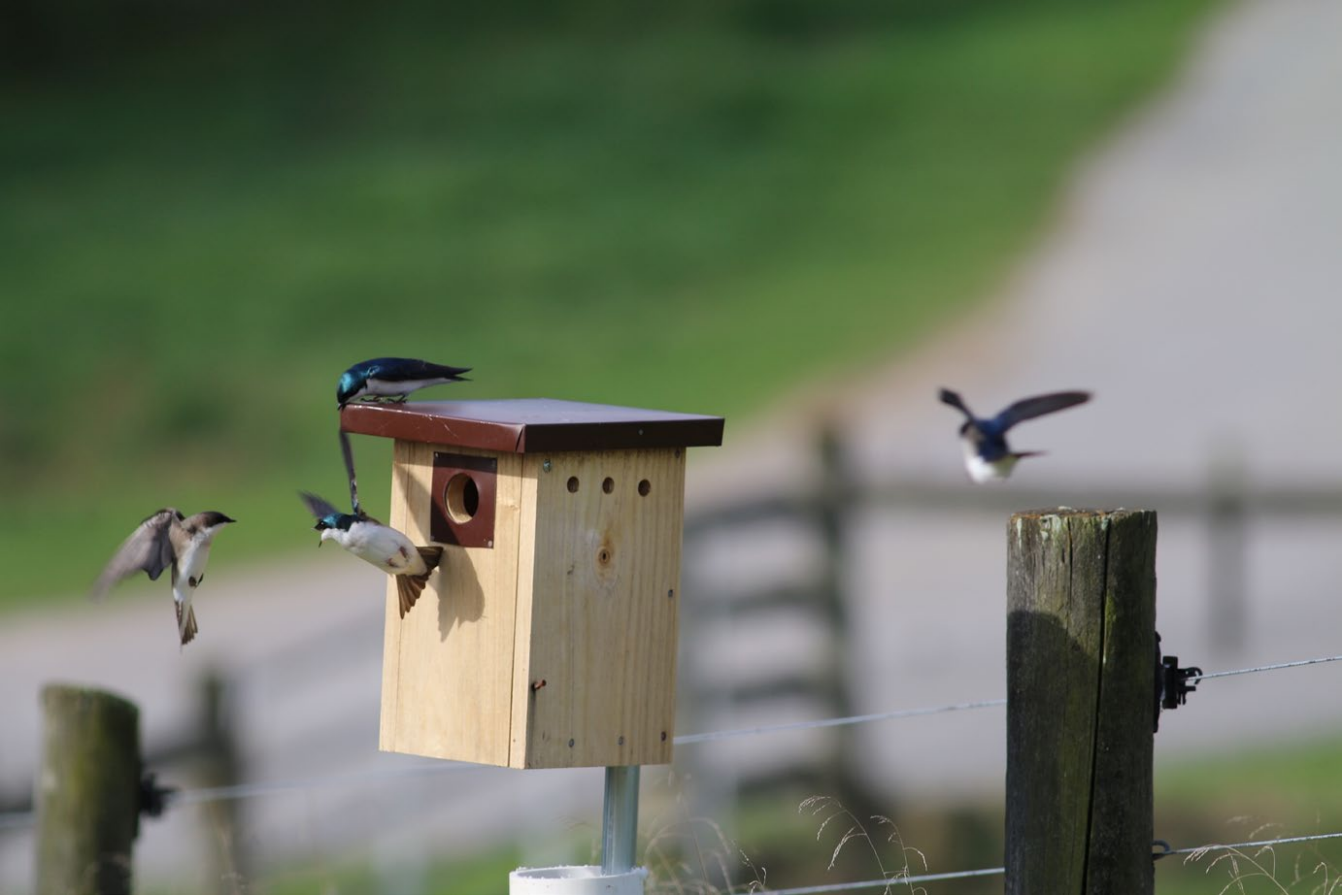
Tree swallows during the breeding season in southwestern Virginia, USA. Photo captured by Ben J. Vernasco

We studied a breeding population of free‐living tree swallows at Virginia Tech's Kentland Farm in Montgomery County, Virginia (37°11′53.6 N, 80°34′58.0 W; 520 m.a.s.l.; Figure 1). In this population, tree swallows breed from late March to early August and use nest boxes that we have set approximately 25 m apart bordering agricultural fields. Tree swallows are socially monogamous, however, both females and males may engage in extra‐pair solicitations, copulations, and fertilizations (Dunn et al., 2009; Lifjeld et al., 1993). In previous studies of other populations, the proportion of tree swallow broods containing extra‐pair young ranged from 68% to 87% (see Table 4 in Conrad et al., 2001, Dunn et al., 1994; Dunn et al., 1994, Lifjeld et al., 1993; Kempenaers et al., 1999). Tree swallows are generally considered to be a single‐brooded species; however, there is some evidence for double‐brooding (e.g., Monroe et al., 2008). In our study population, the majority of females are single‐brooded. The few that lay a second clutch each year do so if their first clutch failed, often due to predation or a cold snap. Only one or two females a year will lay a second clutch after having a successful first clutch (Hernandez, *unpublished data*). All methods in this study were approved by the Institutional Animal Care and Use Committee of Virginia Tech.

### 2.2 Study design

Our goal for the observational study was to assess the relationship between the number of sires per brood and variation in the female's cloacal microbiome. We sampled a total of 388 individuals (71 adult females and 317 nestlings) in 2017 (Table 1). The cloacal bacterial samples from all 71 adult females were used for microbiome analyses. We assessed age based on plumage as first‐time breeding females (or females in their second year, hereafter, “SY” females) had immature brown plumage, while females breeding after their second year (or after second year females, hereafter, “ASY” females) had mature blue plumage (Hussell, 1983). To assess rates of extra‐pair fertilizations (a conservative measure of extra‐pair copulations), 60 of the 71 sampled females and their putative offspring (i.e., 278 nestlings comprising 60 broods) were genotyped for paternity analyses (via DNA extracted from blood). See Table 1 for sample sizes. Each individual was sampled once.

**TABLE 1:** Number of female tree swallows sampled with respect to treatment group, number of sires per brood, age, and year

Number of sires per brood	Observational study	Experimental study		
	No implant	No implant	Blank implant	Estradiol implant
1	12	6	2	1
2	35	3	7	9
3	11	1	5	2
4	2	1	‐	‐
No data				
Predated	‐	‐	‐	2
Abandoned	‐	‐	2	3
Age				
SY	26	2	3	4
ASY	45	9	13	13
Year				
2017	71			
2018		3	3	4
2019		8	13	13

Our goal for the experimental study was to assess the effects of increased sexual activity on the richness and community structure of the female cloacal microbiome. We experimentally administered exogenous estradiol and subsequently assessed nestling paternity to determine whether the treatment affected extra‐pair fertilizations, in addition to overall sexual activity. We included 10 females (*N* = 3 SY, *N* = 7 ASY) and 41 nestlings from 2018, and 34 females (*N* = 7 SY, *N* = 27 ASY) and 112 nestlings from 2019 in this study (Table 1). We were unable to genotype all females and their respective broods due to females abandoning their nests (*N* = 5 in 2019, 3 from estradiol‐implanted and 2 from blank‐implanted females) or nests being predated (*N* = 1 in 2018, *N* = 1 in 2019, both from SY estradiol‐implanted females; Table 1). We considered nests to have been predated if we observed a snake in the box or a ransacked nest, or abandoned if the eggs were present but cold and never hatched. The blood DNA of 37 females and their putative offspring (i.e., 153 nestlings comprising 37 broods) were genotyped for paternity analyses (Table 1). These included 9 females and 41 offspring comprising 9 broods genotyped in 2018, and 28 females and 112 offspring comprising 28 broods genotyped in 2019. We combined the data for 2018 and 2019 seasons for subsequent statistical analyses.

### 2.3 Sample collection

For the observational study, we caught adult and nestling tree swallows in nest boxes from March to July 2017. We determined the pair‐bonded female and male social pair of a nest through observational surveys. Specifically, we assumed that the female found incubating the eggs and the male seen most frequently feeding the subsequently hatched nestlings were the social pair of each nest. We captured and sampled adult females on day six of incubation, adult males on day three of nestling provisioning, and nestlings on day six posthatching. In tree swallows, incubation typically lasts 11–20 days and the nestling period lasts 15–25 days (Winkler et al., 2020), with nestling provisioning occurring from the onset of nestlings hatching to just a few days prior to fledging. To collect cloacal bacteria for microbiome analyses, we gently inserted a sterile swab (PurFlock®, Puritan, USA) approximately 4 mm into the cloaca of adult tree swallows and revolved it once. We used sterile techniques when sampling the cloaca by using new gloves and a new presterilized and presealed swab per bird sampled. To collect blood for paternity analyses and condition assessments, we gently punctured the brachial vein and collected ~100 µl of blood from adults and ~75 µl of blood from nestlings using microhematocrit capillary tubes. Swab and blood samples were stored on ice in the field. Swab samples were frozen upon return to the laboratory (<5 hr postcollection) in a −80°C freezer. Blood samples were centrifuged, and then, plasma and red blood cells were frozen in separate tubes.

For the experimental study, we caught each adult female twice, first during nest building, which is prior to egg‐laying, and again on day six of egg incubation (mean time between sampling breeding periods: 23 days; range: 9–66 days) from March to July 2018 and 2019. Upon each capture, we sampled cloacal bacteria using a sterile swab (PurFlock®, Puritan, USA), collected a blood sample, and took morphometric measurements. The first time we caught each female (i.e., during nest building), we randomly assigned her to one of three groups: a no implant control, a blank implant control, or an estradiol implant. Females in the estradiol treatment group received a silastic implant packed with crystalline estradiol (e.g., Danner et al., 2011), while females in the blank implant group received an empty silastic implant. All implants were ~6 mm in length, sealed at both ends, and were inserted subcutaneously along the female's left flank. We chose to make the first sampling period prior to egg‐laying to allow enough time for the implant to release exogenous hormone (if the female was given an estradiol implant) and thus alter the sexual behavior of the female before laying. Previous work in a temperate‐breeding songbird found that females treated with exogenous estradiol exhibited significantly more solicitation displays compared to control females as soon as 4 days postimplantation (Searcy & Capp, 1997) and as long as 80 days postimplantation (Moore, 1982). The second time we caught each female, we collected all samples and then removed the implant. We chose to take the second sample on day six of egg incubation to decrease the likelihood that the female would abandon the breeding attempt. Adult male and nestlings were captured and sampled as before.

### 2.4 Body condition estimates

To assess body condition, we calculated both a morphological‐ and a physiological‐based index. For the morphological index, we calculated a scaled‐mass index based on a bird's mass and wing length (Peig & Green, 2009). For the physiological index, we measured each bird's hematocrit, the ratio of red blood cell volume to total blood volume (Minias, 2014 but see: Smith & Barber, 2012).

### 2.5 Blood DNA processing

Blood DNA samples were processed in fall 2017 for the observational study and in 2018 and 2019 for the experimental study. To extract DNA from blood samples, we used the DNeasy Blood and Tissue Kit (Qiagen Inc., Valencia, CA, USA). We extended the initial 56°C incubation to 24 hr to optimize the lysing of red blood cells. To perform multiplex PCR‐based analyses of microsatellites, we used the Type‐it Microsatellite PCR Kit (Qiagen Inc., Valencia, CA, USA) and amplified the extracted DNA at eight highly polymorphic microsatellite loci in two multiplexed PCRs (See Table S1). We amplified the extracted DNA at two additional highly polymorphic microsatellite loci (Tal11 and Tal8) in our multiplexed PCRs for the experimental study (Table S2). Forward primers were fluorescently labeled. We modified the Type‐it® Microsatellite PCR Kit protocol to run 12.5 μl reactions. The thermal cycling conditions were as follows: 2 min at 95°C, followed by 28 cycles of 30 s at 95°C, 90 s at 56°C or 58°C, and 30 s at 72°C. A final extension step consisted of 30 min at 60°C. PCR mix A (see Table S1) was run at 56°C, while PCR mix B was run at 58°C. We prepared a 1:10 dilution of each multiplexed PCR product and sent samples to the DNA Analysis Facility on Science Hill at Yale University for genotyping. Genotyping was performed on a 3730xl 96‐Capillary Genetic Analyzer (Thermo Fisher Scientific, Waltham, MA), with DNA fragment sizes estimated using GeneScan™ 500 LIZ® size standard.

We scored alleles using Geneious v.10.2.2 (Biomatters) and then determined the observed and expected heterozygosity of loci using CERVUS (v. 3.0.7; Kalinowski et al., 2007; Marshall et al., 1998). Next, we inferred parentage and sibship using COLONY (v. 2.0.6.5; Wang, 2004; Jones & Wang, 2010). We specified a female and male polygamous mating system, without inbreeding or cloning. We selected a full‐likelihood analysis approach, with a medium run length, and medium likelihood precision. We opted not to have the program update allele frequency, scale sibship, or assume a prior maternal or paternal sibship size distribution. Further, we specified a codominant marker type, 0.001 allelic dropout rate, and 0.001 genotyping error rate for all loci. We also allowed for putative mothers to mismatch offspring at only one locus (Fernando et al., 2001; Ferretti et al., 2016). The allele frequency and inbreeding coefficient for the sampled population was estimated in COLONY based on the genotypes of all sampled individuals. From the COLONY output, we first compared the genetic profiles of nestlings and the social female (putative mother) to confirm maternal parentage. Then, based on the genotypes of the sampled nestlings, the genotype of the sampled mother, and the frequency of alleles of the sampled population, COLONY reconstructed the minimum number of sires (based on genotypes) that would need to exist to account for the allelic variation in nestling genotypes within a brood.

### 2.6 Bacterial DNA processing

We processed bacterial DNA samples as in Hernandez et al. (2020) for bacterial DNA extraction, amplification, and sequencing. Briefly, we extracted DNA from cloacal swabs using the DNeasy Blood and Tissue Kit protocol. We used one kit for the observational study samples and one kit for both years of the experimental study samples. We used the primers 515F and 806R to target the hypervariable V4 region of the 16S rRNA gene for amplification. The 806R primers included uniquely indexed adaptors to allow for multiplexing. We performed PCR in triplicate and ran negative controls without template DNA for each sample. We pooled the triplicate reactions for each sample and visualized the PCR products using agarose gel electrophoresis. Then, we quantified the amplified bacterial DNA in each sample using a Qubit 2.0 Fluorometer and a dsDNA High Sensitivity assay kit. Lastly, we pooled 200 ng DNA from each sample into a single library for sequencing. The library was sequenced using 1 × 250 bp sequencing on the Illumina MiSeq instrument, as described by Caporaso et al. (2012), at the Dana Farber Cancer Institute of Harvard University. Samples collected in 2017 and 2018 were each sequenced in separate runs (2017:71 samples, 2018:20 samples). Samples collected in 2019 were sequenced in two separate runs (34 samples/run), with samples randomly allocated to each run.

We demultiplexed and quality filtered single end reads of raw 16S rRNA amplicon sequences using the Quantitative Insights Into Microbial Ecology (QIIME) 2 (version 2018.11) pipeline (Bolyen et al., 2019). Reads were error corrected and filtered to remove phiX and chimeric reads using DADA2 (Callahan et al., 2016). We specified a truncation quality score of 11, with no end trimming, retaining the full 250bp read length across all samples. After DADA2 filtering, reads per sample for the observational study ranged from 31,252 to 168,791, with a total of 71 samples and 14,111 amplicon sequence variants (ASVs) represented. After DADA2 filtering, we merged the three resulting ASV tables for the experimental study together and the reads per sample ranged from 11 to 121,932, with a total of 88 samples and 20,336 ASVs represented. We next removed ASVs with fewer than 0.01% of the total number of reads (i.e., total frequency fewer than 829 reads in the observational study dataset and 747 in the experimental study dataset) (Bokulich et al., 2013). To taxonomically classify our ASVs, we used a Naïve Bayes classifier that was pretrained on the Silva 132 database 99% ASVs from the 515F/806R region of sequences (Quast et al., 2013) using scikit‐learn 0.20.2 (Pedregosa et al., 2011). We filtered out all ASVs that were taxonomically annotated as Mitochondria, Chloroplast, Eukaryota, and Unassigned. Our final sampling depth for the observational study dataset ranged from 25,159 to 159,867. Based on the alpha rarefaction curve, samples were rarefied to 25,000 to standardize sequencing effort. Our final observational study table contained 71 samples and 527 ASVs. Our final sampling depth for the experimental study dataset ranged from 1,380 to 121,359, with 682 ASVs represented. Based on alpha rarefaction curves, we rarefied samples to 7,400. Our final experimental study table contained 88 samples and 682 ASVs.

### 2.7 Hormone assay

We measured the plasma volume for all blood samples (2017 mean: 26.6 μl, 2018 mean: 33.8 μl, 2019 mean: 34.2 μl) and then extracted the sample with dichloromethane. Then, we quantified the total plasma estradiol concentration (ng/ml) using a direct radioimmunoassay (following Moore et al., 2004; Wingfield et al., 1991), adjusted for individual extraction efficiency (2017:42.1%, 2018:42.1%, 2019:46.1%). We ran samples from 2017 and 2018 in the same assay, and samples from 2019 in a separate assay. To maximize the hormone detection probability, we ran samples in singlet in each assay. The order of individual samples within the assay was randomized. The intra‐assay coefficient of variation, which estimates the variation among standards within an assay, was 14.6%, 14.6%, and 2.63% for 2017, 2018, and 2019, respectively. The assay limit of detection was ~1.3, 1.3, and 0.5 ng/ml in 2017, 2018, and 2019 and was calculated based on individual plasma volumes and extraction efficiency.

### 2.8 Statistical analyses

#### 2.8.1 Observational study

We performed all analyses in R (v. 3.6.1) (R Core Team, 2019). For the microbiome analyses, we assessed shared and unique ASVs across female cloacal bacterial communities. In addition, we determined the most abundant ASVs in the cloaca of each sampled female bird using a relative abundance cutoff of 5%.

To identify the possible predictors associated with the alpha diversity of female cloacal bacterial communities, we used model selection and multimodal inference approaches. Alpha diversity is an estimate of within‐sample diversity (in this case, within an individual bird's cloaca) and is based on ASVs for this study. We calculated three alpha diversity metrics: ASV richness (the total number of taxa), Shannon Index (considers both species abundance and evenness), and Faith's phylogenetic distance (assesses phylogenetic breadth; hereafter, “Faith's PD”). We generated competing linear models that included cloacal microbiome alpha diversity (ASV richness, Shannon Index, Faith's PD) as the response variable, and number of sires per brood (1, 2, 3+) and female age (SY, ASY) as covariates. We then used Akaike's information criterion (AICc) corrected for small sample sizes to compare and rank these competing models (Table S3; Akaike, 1973; Burnham & Anderson, 2002), selecting models with a ∆AICc of <2 as “better fit” models. We incorporated covariates from the highest ranked models into our assessments of cloacal bacterial community diversity; summaries of competing “best fit” models (∆AICc of <2) are presented in Table S4. Lastly, we used ggplot (“ggplot2”:*ggplot*; Wickham, 2016) for visualizations.

To assess cloacal bacterial beta diversity or whether the variation in cloacal bacterial community structure differed among females with respect to age and number of sires per brood, we performed nonparametric, permutational multivariate analyses of variance (PERMANOVA; “vegan”: *adonis*) based on 999 permutations (Anderson, 2001; McArdle & Anderson, 2001). Beta diversity is an estimate of variation in community structure among samples (in this case, among birds) and was computed based on both Bray–Curtis and Jaccard distance metrics. While Bray–Curtis and Jaccard both evaluate count‐based data, Bray–Curtis evaluates relative abundances, while Jaccard evaluates presence–absence. We compared the multivariate homogeneity of group dispersions (“vegan”: *betadisper*) for sampled females with respect to age and number of sires per brood using a permutation test (“vegan”: *permutest*) (Anderson, 2006; Anderson et al., 2006). Finally, we used a nonmetric multidimensional scaling (NMDS; Kruskal, 1964a, 1964b) plot to visualize the dissimilarity distances among females.

To identify the most relevant predictors associated with the body condition indices of females, we used the same model selection and AICc comparisons as for the alpha diversity analyses above. Briefly, we generated linear models that included body condition (scaled‐mass index, hematocrit) as the response with both number of sires per brood and female age as covariates, and then used AICc to compare and rank the models (Table S5). We incorporated covariates from the highest ranked models into our assessments of condition. We also generated linear models that included the alpha diversity metric (ASV richness, Shannon Index, or Faith's PD) as the response variable and the host body condition as the predictor variable. To assess whether the variation in cloacal bacterial community structure differed among females with respect to body condition, we performed PERMANOVAs and compared the multivariate homogeneity of group dispersions as before.

To assess whether female age or the number of sires per brood had an effect on a female's reproductive success, we assessed average brood mass (i.e., average mass of all nestlings within a brood), hatch success, and fledging success. For each model, we set the reproductive success metric as the response variable. For average brood mass, we chose to fit a linear model (“stats”: *lm*) with female age (or number of sires per brood) and nestling age at sampling as predictor variables. For hatch success, we fit a generalized linear model with a beta distribution (“betareg”: *betareg*) and included female age or number of sires per brood as the predictor variable and weighted the model by the total number of eggs laid (mean: 5 eggs; range: 3–6 eggs). We transformed the response variable (i.e., hatch success) using the transformation recommended by the R documentation for this function, because the data included 0 and 1. The transformation recommended is as follows: (*y*(*n*−1) + 0.5)/*n*)), where *n* is the sample size (Cribari‐Neto & Zeileis, 2009; Smithson & Verkuilen, 2006). For fledging success, we fit a linear model and included female age or number of sires per brood as the predictor variable.

To assess whether the number of sires per brood was influenced by circulating estradiol concentrations and female age, we used model selection and multimodal inference statistical approaches. We generated competing linear models that included the number of sires per brood as the response variable and estradiol concentrations (natural log‐transformed) and female age as predictor variables (Table S9). We removed 8 samples for which there was less than 10 μl plasma used in the radioimmunoassay, since the volume was too low to effectively quantify the estradiol concentration. Then, we used AICc to compare and rank our competing models (Akaike, 1973; Burnham & Anderson, 2002). We incorporated covariates from the highest ranked models into our assessments of circulating estradiol concentrations.

#### 2.8.2 Experimental study

We assessed the clutch and brood sizes for all sampled females. To determine whether clutch or brood size varied among females in different treatment groups, we ran ANOVA models with clutch size or brood size as the response and female treatment group as the predictor, followed by a Tukey's post hoc test to assess significant differences between groups. We also ran an ANOVA model with the number of sires per brood (1, 2, 3, 4) as the response and female treatment group as the predictor. Note that we set the number of sires per brood as a continuous response variable when set as the response but consider the number of sires per brood as a categorical variable when set as a predictor elsewhere.

To confirm whether the administration of exogenous estradiol (via the estradiol implant) had an effect on circulating estradiol concentrations in females, we generated a linear model with estradiol concentrations (natural log‐transformed) as the response variable and treatment group (blank implant, estradiol implant), sampling year (2018, 2019), and the number of days a female was implanted (mean: 23 days; range: 9–66 days) as predictor variables. Note that we only included samples taken during incubation for this analysis and that we only consider the blank implant as the control, since it is the true control for the estradiol implant treatment.

We assessed shared and unique ASVs across female cloacal bacterial communities. In addition, we determined the most abundant ASVs in the cloaca of each sampled female using a relative abundance cutoff of 5%.

We calculated these three alpha diversity metrics (ASV richness, Shannon Index, and Faith's PD) for each female for both sampling events (i.e., nest building and incubation) and then calculated the change in each metric. To assess whether treatment group had an effect on the change in cloacal bacterial diversity, we used model selection and a multimodal inference approach. We generated competing linear models for each diversity metric: ASV richness, Shannon Index, and Faith's PD. For each of the three linear models, we set change in the diversity metric as the response variable and treatment (no implant, blank implant, estradiol implant), year of sampling (2018, 2019), and the number of days a female was implanted (mean: 23 days; range: 9–66 days) as possible explanatory variables (Table S11). We did not include female age in the models because our ability to detect an effect was weak given the low sample sizes (see Table 1). We used ggplot (“ggplot2”:*ggplot*; Wickham, 2016) for visualizations.

To determine whether there was an overall change in female cloacal bacterial diversity between breeding stages regardless of treatment, we fit linear mixed‐effect models and set the diversity metric as the response variable and breeding stage (nest building, incubation) as the explanatory variable with the bird's unique band ID included as a random effect.

To assess whether the variation in the structure of cloacal bacterial communities differed among females, we performed PERMANOVA analyses (“vegan”: *adonis*) based on 999 permutations (Anderson, 2001; McArdle & Anderson, 2001). We set the distance metric (Bray–Curtis, Jaccard) as the response variable and included treatment group, breeding stage, year of sampling, and the number of days implanted as explanatory variables, with an interaction between treatment group and breeding stage. We also controlled for repeated measures in the dataset by including each bird's unique band ID in the “strata” argument of the PERMANOVA model. We compared the multivariate homogeneity of group dispersions (“vegan”: *betadisper*) for sampled females with respect to treatment and sampling event using permutation tests (“vegan”: *permutest*) (Anderson, 2006; Anderson et al., 2006). We performed permutation tests to assess the homogeneity of group dispersions of cloacal bacterial communities per treatment before and after implantation. Finally, we used a NMDS (Kruskal, 1964a, 1964b) plot to visualize the dissimilarity distances between sampled females.

To assess whether treatment group had an effect on a female's condition, we fit a linear model for each condition index. For each model, we set the condition metric as the response variable (scaled‐mass index, hematocrit) and included treatment group, year of sampling, and the number of days implanted as predictor variables. The variable “Year” was not included in models for hematocrit because we did not have data on hematocrit for 2018. In the case of treatment significantly varying with a condition metric, we performed statistical comparisons among treatments (“emmeans”: *contrast*) to compare the hematocrit of females in different treatment groups. Further, to assess whether microbiome diversity and/or community structure varied with host condition, we ran linear models and performed PERMANOVAs as before.

To assess whether treatment group had an effect on a female's reproductive success, we assessed average brood mass, hatch success, and fledging success as for the observational study analyses, with a few changes. For all three models, we set female treatment as the predictor and we performed statistical comparisons among treatments (“emmeans”: *contrast*) to compare the reproductive success of females in different treatment groups.

## 3 RESULTS

### 3.1 Observational study

The average clutch size was 5.2 ± 0.09 eggs (mean ± *SEM*, *N* = 71 clutches, range = 3–6 eggs), and the average brood size was 4.5 ± 0.13 nestlings (mean ± *SEM*, *N* = 71 broods, range = 2–6 nestlings) for the sampled population.

We identified 527 ASVs across 71 individual female cloacal bacteria samples. The families with a mean relative abundance ≥5% included Corynebacteriaceae (relative abundance 23%, phylum Actinobacteria), Enterobacteriaceae (22%, Proteobacteria), Enterococcaceae (7%, Firmicutes), and Micrococcaceae (6%, Actinobacteria). Families within the phyla Bacteroidetes, Chlamydiae, Chloroflexi, Cyanobacteria, Fusobacteria, Deinococcus‐Thermus, Epsilonbacteraeota, FBP, Tenericutes, and an unidentified phylum comprised the remaining ~42%. More specifically, the ASVs with the highest relative abundance (all identified down to genus) included Corynebacterium 1 (22%), Escherichia‐Shigella (16%), Enterococcus (4%), Rothia (4%), and Exiguobacterium (3%). The most prevalent ASVs (identified down to genus) were present in 90% of females and included Allorhizobium‐Neorhizobium‐Pararhizobium‐Rhizobium, Corynebacterium 1, Escherichia‐Shigella, Exiguobacterium, Rothia, and Sphingomonas. There was an average (± standard deviation) of 126 ± 66 ASVs/female (range: 13–281).

**FIGURE 2:**
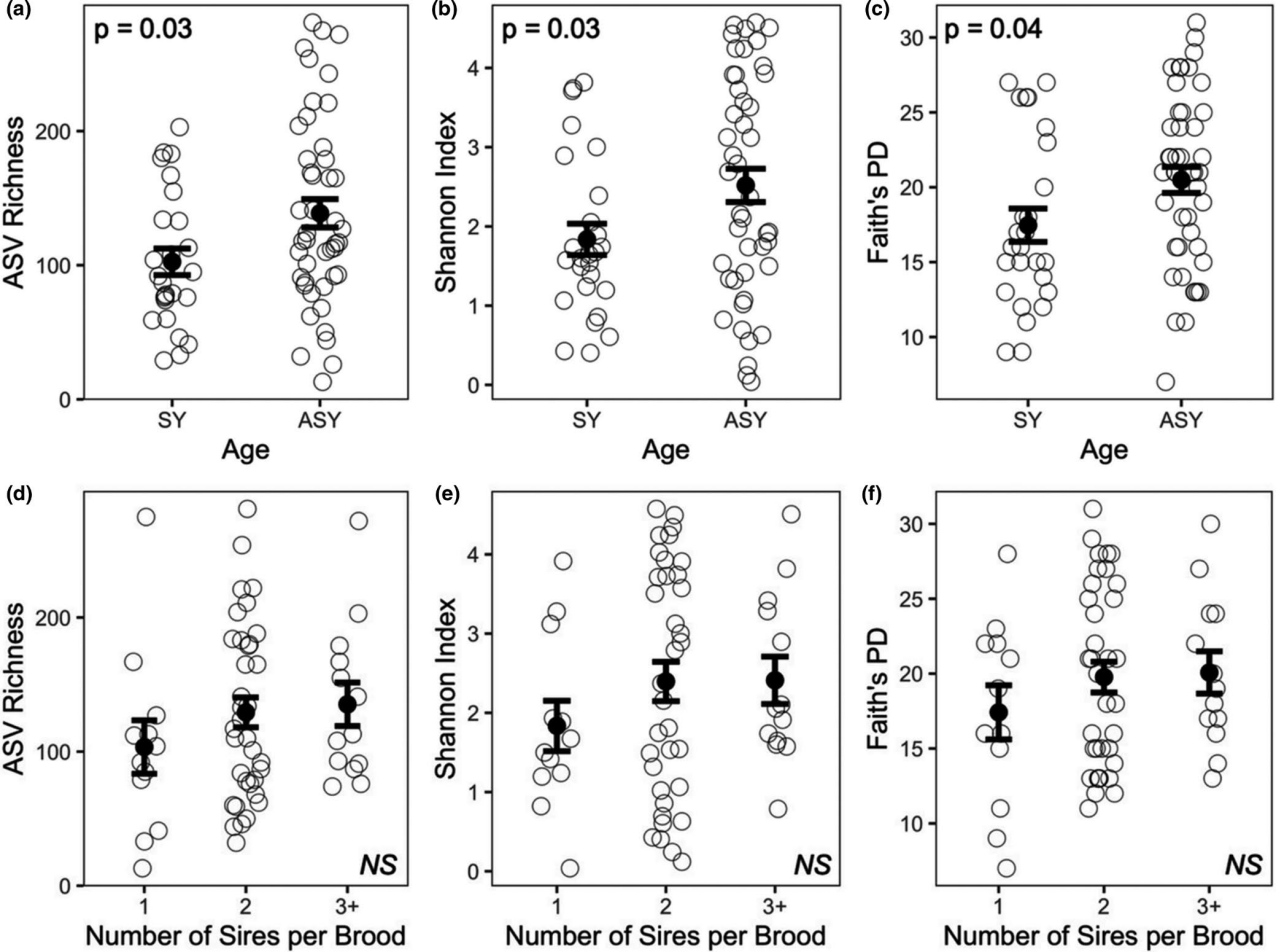
ASV richness (a, d), Shannon Index (b, e), and Faith's phylogenetic distance (c, f) of bacterial amplicon sequence variants (ASVs) sampled from the cloacae of female tree swallows with respect to female age (a–c) and the number of sires (d–f) per brood. ‘SY’ refers to Second Year females, that is, females sampled during their first breeding season, while ‘ASY’ refers to After Second Year females, that is, females sampled after their first breeding season. Each point represents an individual female. Sample sizes include *n* = 26 (SY) and *n* = 45 (ASY) females for panels a–c and *n* = 12 (1 sire), *n* = 35 (2 sires), and *n* = 13 (3+ sires) for panels d–f. Mean ± standard error is shown. *p*‐values are based on linear models

Female age, but not number of sires per brood, was significantly related to ASV richness, the Shannon Index, and Faith's PD (Tables S3 and Table S4). More specifically, female age was positively associated with ASV richness (*b* = 36.2, *SE* = 15.8, *p* = .03), Shannon Index (*b* = 0.68, *SE* = 0.32, *p* = .03), and Faith's PD (*b* = 3.0, *SE* = 1.4, *p* = .04; Figure 2).

**FIGURE 3:**
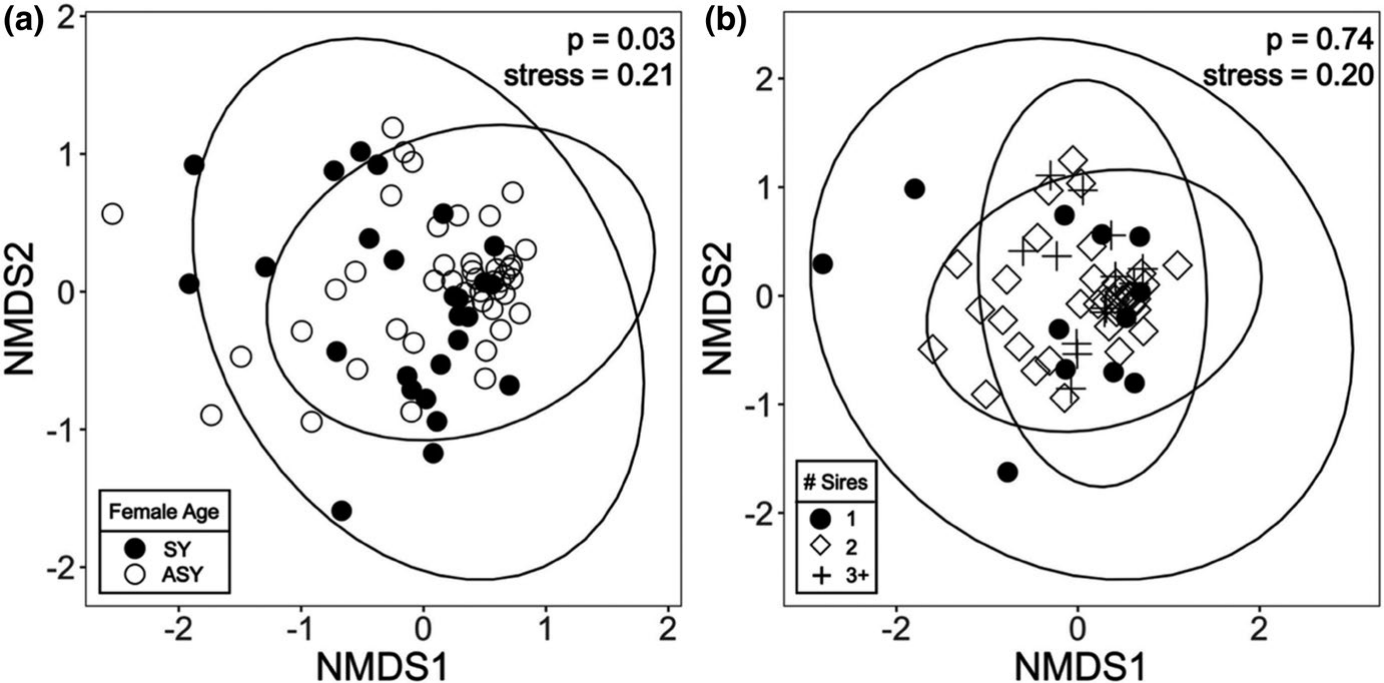
Cloacal bacterial beta diversity (nonmetric multidimensional scaling plot, NMDS, based on Bray–Curtis dissimilarity) of adult tree swallows with respect to (a) female age and (b) number of sires per brood. Each point represents the cloacal bacterial community of one female. In (a), closed circles = SY females, open circles = ASY females. In (b), closed circles = broods with 1 sire, open diamonds = broods with 2 sires, cross = broods with 3+ sires. Sample sizes include *n* = 71 for panel a and *n* = 60 for panel b. NMDS based on Jaccard dissimilarity (not pictured here) is similar. *p*‐values are based on PERMANOVA models

Female age also explained significant variation in the structure of cloacal bacterial communities (Bray–Curtis: *pseudo‐F*_1,69_ = 2.3, *p* = .03; Jaccard: *pseudo‐F*_1,69_ = 2.0, *p* = .02), though the effect was relatively weak (Bray–Curtis: *R* ^2^ = 0.03; Jaccard: *R* ^2^ = 0.03) (Figure 3a). The dispersion of female cloacal bacterial communities did not differ significantly based on female age (Bray–Curtis: *pseudo‐F*_1,69_ = 0.62, *p* = .43; Jaccard: *pseudo‐F*_1,69_ = 0.89, *p* = .35). Females did not significantly differ in the structure of cloacal bacterial communities when considering their number of sires per brood (Bray–Curtis: *pseudo‐F*_2,57_ = 0.78, *R* ^2^ = 0.03, *p* = .74; Jaccard: *pseudo‐F*_2,57_ = 0.83, *R* ^2^ = 0.03, *p* = .75) (Figure 3b). The dispersion of female cloacal bacterial communities did not differ significantly based on number of sires (Bray–Curtis: *pseudo‐F*_2,57_ = 0.52, *p* = .60; Jaccard: *pseudo‐F*_2,57_ = 0.50, *p* = .61).

We found some support for a relationship between scaled‐mass index and the number of sires per brood, but not between scaled‐mass index and female age (Tables S5, Table S6, Table S7). More specifically, the difference in scaled‐mass index between females with one sire per brood compared to two sires per brood was significant (1–2: *b* = −1.8, *SE* = 0.66, *p* = .03). We also found no evidence for a relationship between hematocrit and either number of sires per brood or female age (Tables S5, Table S6, Table S7).

Additionally, we found no relationship between either scaled‐mass index or hematocrit and cloacal microbiome alpha diversity (*SMi*, ASV richness: *b* = 3.3, *SE* = 3.9, *p* = .40; Shannon Index: *b* = 0.02, *SE* = 0.08, *p* = .76; Faith's PD: *b* = 0.16, *SE* = 0.35, *p* = .65; *Hematocrit*, ASV richness: *b* = 70.3, *SE* = 260, *p* = .79; Shannon Index: *b* = 3.8, *SE* = 4.9, *p* = .44; Faith's PD: *b* = 13.0, *SE* = 23.0, *p* = .57). There was no relationship between either scaled‐mass index or hematocrit and cloacal microbiome structure (*SMi*, Bray–Curtis: *pseudo‐F*_1,68_ = 0.61, *R* ^2^ = 0.01, *p* = .84; Jaccard: *pseudo‐F*_1,68_ = 0.66, *R* ^2^ = 0.01, *p* = .90; *Hematocrit*, Bray–Curtis: *pseudo‐F*_1,65_ = 0.55, *R* ^2^ = 0.01, *p* = .89; Jaccard: *pseudo‐F*_1,65_ = 0.66, *R* ^2^ = 0.01, *p* = .90).

Hatch success, but not average brood mass or fledging success, significantly varied with female age (Table S8). More specifically, broods of ASY females had significantly lower hatch success compared to broods of SY females (*b* = −0.59, *SE* = 0.11, *p* < .001). There were no relationships between any of these three reproductive success proxies and the number of sires per brood (Table S8).

We found no relationship between the number of sires per brood and circulating estradiol concentrations (Table S9, Table S10), even when considering female age (Table S9, Table S10).

### 3.2 Experimental study

In 2018, the average clutch size was 5.0 ± 0.15 eggs (mean ± *SEM*, *N* = 10 clutches, range = 4–6 eggs) and the average brood size was 4.1 ± 0.50 nestlings (mean ± *SEM*, *N* = 10 broods, range = 0–5 nestlings) for the sampled population. In 2019, the average clutch size was 5.1 ± 0.21 eggs (mean ± *SEM*, *N* = 34 clutches, range = 2–8 eggs) and the average brood size was 3.3 ± 0.37 nestlings (mean ± *SEM*, *N* = 34 broods, range = 0–7 nestlings) for the sampled population. Neither clutch size nor brood size significantly differed among females based on treatment group (*ANOVA*, clutch size*: F*_2,41_ = 1 = 0.9, *p* = .41, brood size: *F*_2,41_ = 2.2, *p* = .13). The number of sires per brood was not statistically different with respect to treatment group (*ANOVA, F*_2,34_ = 1.3, *p* = .28); the mean number of sires per brood was 1.7 for nonimplanted females, 2.2 for blank‐implanted females, and 2.1 for estradiol‐implanted females.

Exogenous estradiol administration (via the estradiol implant) did have an effect on circulating estradiol concentrations (*b* = 0.69, *SE* = 0.29, *p* = .03), with circulating estradiol concentrations higher in estradiol‐implanted females compared to blank‐implanted females during incubation. Sampling year also had a significant effect on circulating estradiol concentrations (*b* = −0.97, *SE* = 0.34, *p* = .01), with females sampled in 2019 exhibiting lower estradiol concentrations compared to females sampled in 2018 during incubation. There was no effect of the number of days a female was implanted on circulating estradiol concentrations (*b* = −0.002, *SE* = 0.01, *p* = .86).

We identified 682 ASVs across 88 female cloacal bacteria samples. During nest building, the families with a mean relative abundance ≥5% included Rhizobiaceae (relative abundance 14%, phylum Proteobacteria), Corynebacteriaceae (14%, Actinobacteria), Sphingomonadaceae (9%, Proteobacteria), Enterobacteriaceae (7%, Proteobacteria), Burkholderiaceae (6%, Proteobacteria), and Mycoplasmataceae (5%, Tenericutes). Families within the phyla Acidobacteria, Bacteroidetes, Chlamydiae, Chloroflexi, Cyanobacteria, Deinococcus‐Thermus, Dependentiae, FBP, Firmicutes, Opisthokonta, Planctomycetes, Verrucomicrobia, and an unidentified phylum comprised the remaining ~45%. More specifically, the ASVs with the highest relative abundance (all identified down to genus, except for the last one which was identified down to family) included Corynebacterium 1 (14%), Allorhizobium‐Neorhizobium‐Pararhizobium‐Rhizobium (7%), Escherichia‐Shigella (4%), Sphingomonas (3%), and Rhizobiaceae (3%). The most prevalent ASVs (present in 90% of females and identified down to genus) were Allorhizobium‐Neorhizobium‐Pararhizobium‐Rhizobium, Flavobacterium, Pantoea, and Sphingomonas. There was an average (± standard deviation) of 135 ± 52 ASVs per female (range: 49–274). During incubation, the families with a mean relative abundance ≥5% included Enterobacteriaceae (20%, Proteobacteria), Corynebacteriaceae (16%, Actinobacteria), Enterococcaceae (7%, Firmicutes), Mycoplasmataceae (7%, Tenericutes), and Rhizobiaceae (6%, Proteobacteria). Families within the phyla Acidobacteria, Bacteroidetes, Chlamydiae, Chloroflexi, Cyanobacteria, Deinococcus‐Thermus, Dependentiae, FBP, Opisthokonta, Planctomycetes, Verrucomicrobia, and an unidentified phylum comprised the remaining ~44%. More specifically, the ASVs with the highest relative abundance (all identified down to genus) included Corynebacterium 1 (16%), Escherichia‐Shigella (14%), Catellicoccus (6%), Allorhizobium‐Neorhizobium‐Pararhizobium‐Rhizobium (3%), and Ureaplasma (3%). The most prevalent ASVs (present in 90% of females and identified down to genus) were Corynebacterium 1, Escherichia‐Shigella, Flavobacterium, and Sphingomonas. There was an average (± standard deviation) of 116 ± 53 ASVs per female (range: 19–226).

There was no significant relationship between the change in female cloacal bacteria alpha diversity (ASV richness, Shannon Index, or Faith's PD) and treatment group (Tables S11, Table S12). There was a positive relationship between the number of days a female was implanted (i.e., time between the two sampling periods) and both ASV richness (*b* = 1.4, *SE* = 0.58, *p* = .02) and Faith's PD (*b* = 0.17, *SE* = 0.1, *p* = .04). And there was a negative relationship between the change in Faith's PD and sampling year (*b* = −5.3, *SE* = 2.5, *p* = .04).

**FIGURE 4:**
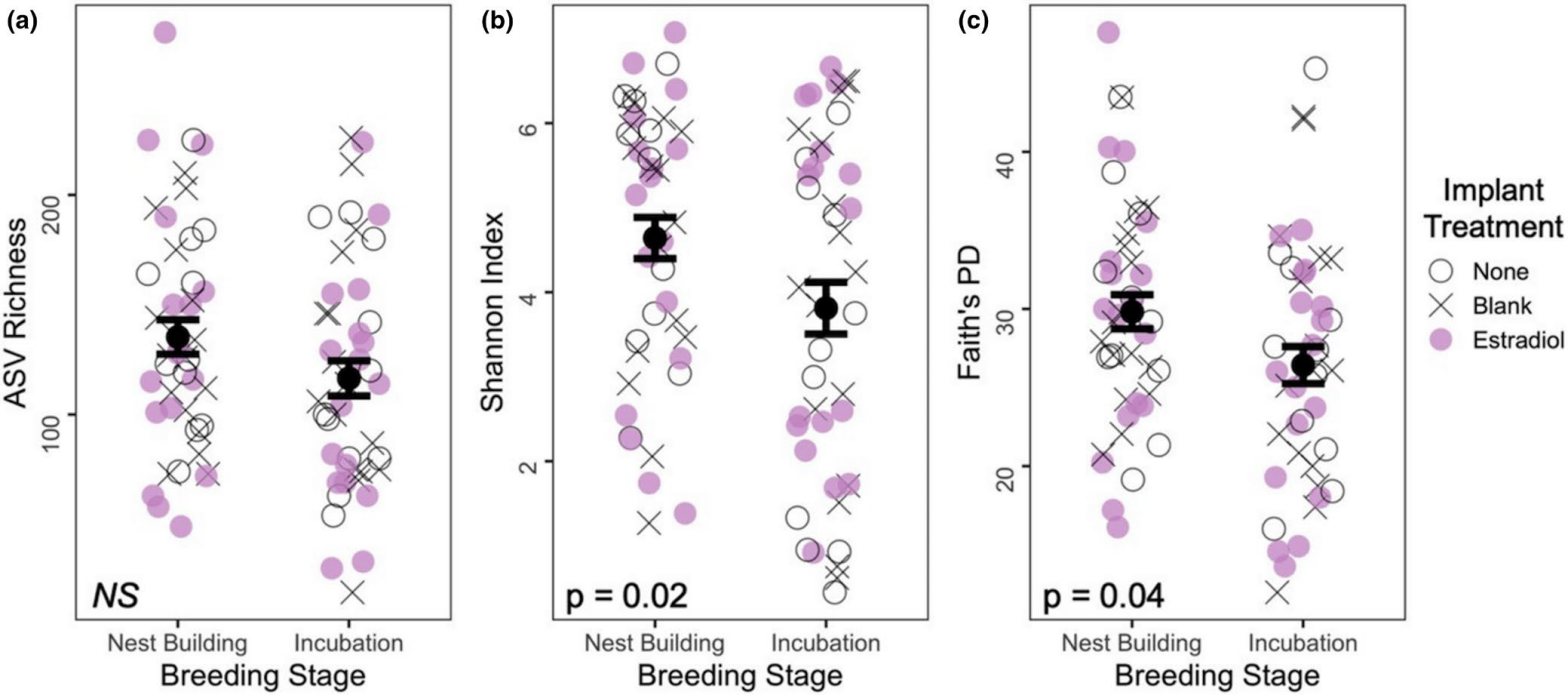
Alpha diversity metrics for bacterial amplicon sequence variants (ASVs) sampled from the cloacae of female tree swallows taken during two breeding stages (Nest Building, Incubation). ASV richness (a), Shannon Index (b), and Faith's phylogenetic diversity (c) indices were calculated. The first sampling time point (Nest Building) refers to initial capture and implantation, while the second sampling time point (Incubation) refers to the second capture event when the implant was removed. Point color and shape represent female treatment group: no implant control = open circle, blank implant = X, estradiol implant = pink filled circle. A total of 44 females were sampled during nest building and the same females were sampled again during incubation. Sample sizes include *n* = 10 females in 2018 and *n* = 34 females in 2019. Mean ± *SEM* are shown. *p*‐values are based on linear mixed‐effect models

Regardless of treatment, female cloacal bacterial diversity decreased between nest building and incubation (Figure 4). More specifically, there was a significant decrease in Shannon Index (*b* = −0.83, *SE* = 0.33, *p* = .02) and in Faith's PD (*b* = −3.4, *SE* = 1.6, *p* = .04), and a trending decrease in ASV richness between nest building and incubation (*b* = −18.8, *SE* = 11.1, *p* = .10).

**FIGURE 5:**
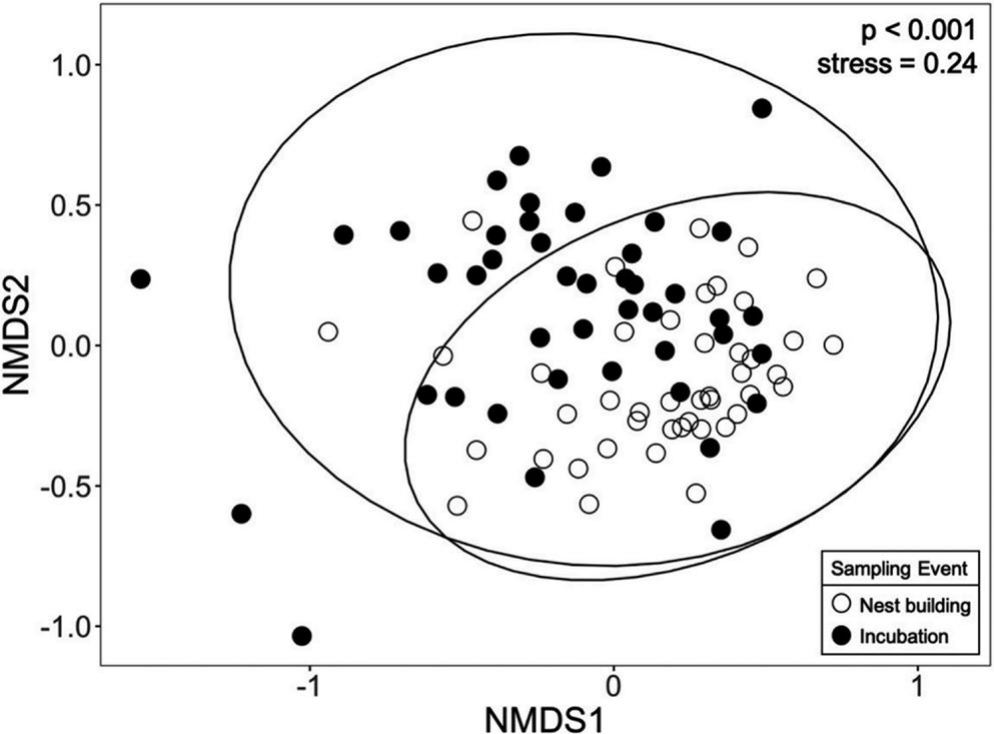
Cloacal bacterial beta diversity (nonmetric multidimensional scaling plot, NMDS based on Bray–Curtis dissimilarity) of female tree swallows at two breeding stages (nest building, incubation). Each circle represents an individual female. Open circles = females sampled during nest building, filled circles = females sampled during incubation. Circles closer together indicate individuals with more similar cloacal bacterial community composition. Sample sizes include *n* = 44 females per sampling period. NMDS based on Jaccard dissimilarity (not pictured here) is similar. *p*‐value is based on a PERMANOVA model. See Figure S1 for an NMDS connecting individual female points

There was a significant difference in cloacal bacterial community structure between nest building and incubation (Bray–Curtis: *pseudo‐F*_1,80_ = 3.4, *R* ^2^ = 0.04, *p* < .001; Jaccard: *pseudo‐F*_1,80_ = 2.4, *R* ^2^ = 0.03, *p* < .001), though the effect was relatively weak (Figure 5). There was no significant difference in cloacal bacterial community structure when considering treatment group (Bray–Curtis: *pseudo‐F*_2,80_ = 1.4, *R* ^2^ = 0.03, *p* = .31; Jaccard: *pseudo‐F*_2,80_ = 1.2, *R* ^2^ = 0.03, *p* = .32) or the number of days a female was implanted (Bray–Curtis: *pseudo‐F*_1,80_ = 2.0, *R* ^2^ = 0.02, *p* = .16; Jaccard: *pseudo‐F*_1,80_ = 1.5, *R* ^2^ = 0.02, *p* = .17). There were trends in cloacal bacterial community structure when considering year (Bray–Curtis: *pseudo‐F*_1,80_ = 2.1, *R* ^2^ = 0.02, *p* = .08; Jaccard: *pseudo‐F*_1,80_ = 1.7, *R* ^2^ = 0.02, *p* = .07) and the interaction between breeding stage and treatment (Bray–Curtis: *pseudo‐F*_2,80_ = 1.2, *R* ^2^ = 0.02, *p* = .08; Jaccard: *pseudo‐F*_2,80_ = 1.1, *R* ^2^ = 0.02, *p* = .08), though the effect sizes were weak. The dispersion of female cloacal bacterial communities was significantly different when considering treatment (Bray–Curtis: *pseudo‐F*_2,85_ = 3.7, *p* = .03; Jaccard: *pseudo‐F*_2,85_ = 4.7, *p* = .01), breeding stage (Bray–Curtis: *pseudo‐F*_1,86_ = 5.9, *p* = .02; Jaccard: *pseudo‐F*_1,86_ = 4.2, *p* = .04), sampling year (Bray–Curtis: *pseudo‐F*_1,86_ = 11.2, *p* = .002; Jaccard: *pseudo‐F*_1,86_ = 13.4, *p* < .001), and the number of days implanted (Bray–Curtis: *pseudo‐F*_24,63_ = 2.4, *p* = .004; Jaccard: *pseudo‐F*_24,63_ = 6.1, *p* < .001). Females among treatment groups did not differ in cloacal bacterial community structure during nest building nor during incubation when considered separately (Tables S13, Table S14).

Estradiol‐implanted females had significantly lower hematocrit compared to blank‐implanted females (Blank‐Estradiol: *b* = 0.03, *SE* = 0.01, *p* = .03). The difference in hematocrit of nonimplanted and estradiol‐implanted females (None‐Estradiol: *b* = 0.02, *SE* = 0.01, *p* = .15) or of nonimplanted and blank‐implanted females (None‐Blank: *b* = −0.01, *SE* = 0.01, *p* = .85) was not statistically significant. There was also no statistical difference when comparing the hematocrit of females between treatment groups prior to implantation (None‐Blank: *b* = 0.01, *SE* = 0.01, *p* = .86; None‐Estradiol: *b* = 0.01, *SE* = 0.01, *p* = .61; Blank‐Estradiol: *b* = 0.01, *SE* = 0.01, *p* = .87). The difference in female scaled‐mass index between sampling years was statistically significant, with females sampled in 2019 exhibiting lower scaled‐mass index on average compared to females sampled in 2018 (*b* = −2.1, *SE* = 0.84, *p* = .02). There was no relationship between scaled‐mass index and treatment (None‐Blank: *b* = 0.48, *SE* = 0.91, *p* = .60; None‐Estradiol: *b* = 0.74, *SE* = 0.92, *p* = .43) or the number of days a female was implanted (*b* = −0.04, *SE* = 0.03, *p* = .14).

We found no relationship between cloacal microbiome diversity and either scaled‐mass index (ASV richness: *b* = −0.62, *SE* = 3.4, *p* = .86; Shannon Index: *b* = 0.15, *SE* = 0.13, *p* = .25; Faith's PD: *b* = −0.04, *SE* = 0.50, *p* = .94) or hematocrit (ASV richness: *b* = 280, *SE* = 340, *p* = .42; Shannon Index: *b* = 12, *SE* = 13, *p* = .38; Faith's PD: *b* = 67, *SE* = 47, *p* = .16). Nor was there a relationship between cloacal microbiome structure and either scaled‐mass index (Bray–Curtis: *pseudo‐F*_1,42_ = 1.1, *R* ^2^ = 0.03, *p* = .28; Jaccard: *pseudo‐F*_1,42_ = 1.1, *R* ^2^ = 0.03, *p* = .23) or hematocrit (Bray–Curtis: *pseudo‐F*_1,30_ = 0.76, *R* ^2^ = 0.02, *p* = .74; Jaccard: *pseudo‐F*_1,30_ = 0.82, *R* ^2^ = 0.03, *p* =.76).

Hatch success, but not average brood mass or fledging success, varied significantly with female treatment group (Table S15). More specifically, estradiol‐implanted females had significantly lower hatch success compared to nonimplanted females (None‐Estradiol: *b* = 0.17, *SE* = 0.05, *p* = .001). There was a trending difference between the hatch success of estradiol‐ and blank‐implanted females (Blank‐Estradiol: *b* = 0.10, *SE* = 0.05, *p* = .09), but not between non‐ and blank‐implanted females (None‐Blank: *b* = 0.07, *SE* = 0.05, *p* = .24).

## 4 DISCUSSION

In this study, we combined robust observational and experimental approaches to test the hypotheses that the number of sires per brood (observational study) and increased sexual activity of females via experimentally elevated estradiol concentrations (experimental study) significantly influence the richness and community structure of the female cloacal microbiome. We did not find support for either hypothesis within the context of one breeding season. The lack of a significant effect may be due to (1) the duration of the study being too short to detect a relationship between sexual behavior and the cloacal microbiome and/or (2) the limitation of using the number of sires per brood as a proxy for the number of mates per female in a system where the rates of extra‐pair activity are high. In contrast to these results, our findings that female age positively predicted cloacal microbiome diversity (ASV richness, Shannon Index, Faith's PD) and influenced shifts in bacterial community structure (based on Bray–Curtis and Jaccard distance metrics) may suggest that, over longer time periods, multiple breeding attempts could alter the cloacal microbiome in distinct ways. In addition, our study is one of the few studies to document within‐individual changes in the cloacal microbiome within one breeding season, thus highlighting how dynamic the cloacal microbiome can be on a short timescale. Taken together, our results contribute to our understanding of the role that host life history and behavior play in shaping the cloacal microbiomes of wild birds.

We found support for our prediction that older females would have higher richness and a differently structured cloacal microbiome compared to younger females, which may be due to higher lifetime mating opportunities and/or competitive mating advantages associated with age. Older females have presumably secured more copulations across multiple breeding seasons, which could increase exposure to bacteria as social monogamy is probably only seasonal in this species (Lifjeld et al., 1993), though the same pair may mate in subsequent breeding seasons due to high philopatry rates (*ITM, personal observation*; Winkler et al., 2004). Older females may also have a competitive advantage over first‐time breeding females and mate assortatively with older, more experienced males (Bitton et al., 2008; Ferrer & Penteriani, 2003; Johnstone et al., 1996; Robertson & Rendell, 2001), or be better able to capitalize on extra‐pair mating opportunities (Bouwman & Komdeur, 2005). If an age‐assortative mating scenario is true in this tree swallow system, then we would predict older females to exhibit higher cloacal bacterial richness due to a combination of mating with more males and mating with older males that have in turn mated with more females previously. Though we do not have the necessary age data for males to be able to assess whether the older females we sampled were socially paired to older males, tree swallows in other populations consistently mate assortatively by age (Bitton et al., 2008; Robertson & Rendell, 2001). Further, while our study assessed the number of mates per female using the number of sires per brood as a proxy, we did not consider the number of mates of the socially partnered male. In great tits (*Parus major*), males socially paired to older females secured higher rates of extra‐pair paternity (Roth et al., 2019), potentially transmitting bacteria acquired from those extra‐pair copulations back to their social partners. There is also a positive relationship between male age and extra‐pair paternity, as evidenced in two meta‐analyses incorporating studies done across passerine species (Cleasby & Nakagawa, 2012; Hsu et al., 2015). Males with many partners could alter the microbiome of their social partners, but high rates of extra‐pair copulations could also increase mixing and homogeneity of the cloacal microbiome across the entire population.

Additional nonmating‐related processes that may explain age‐related changes in the microbiome include changes in host life history, behavior, immune function, resource demands, and physiology that may occur with aging. Several studies have compared the cloacal microbiome between young and adults and found that the cloacal microbiome, and gastrointestinal tract more broadly, of the young is typically comprised of transient microbes, while that of the adults is comprised of a relatively more stable community of microbes (e.g., van Dongen et al., 2013, Kohl et al., 2018, but also Burns et al., 2016, Trosvik et al., 2010). It is possible that the increased stability of these communities with aging is due in part to the development of the host immune system and increased selection for particular microbes and microbial communities over time (Burns et al., 2016; Kohl et al., 2018; McFall‐Ngai et al., 2013). Investment in immune function can change as host resource demands change over time (Cichoń et al., 2003; Lavoie, 2005; Lozano & Lank, 2003; Saino et al., 2003). For example, a host's investment in survival and reproduction is expected to shift over the course of an animal's life, and such shifts in resource investment may reflect or be reflected by shifts in microbiome alpha diversity and community structure (McFall‐Ngai et al., 2013). Given that investment into both the immune response, survival overall, and reproduction may be physiologically demanding in different ways, changes in physiological function to meet these diverse investment requirements may also reflect changes in the host‐associated microbiome (Ley et al., 2008).

We found a significant decrease in within‐individual cloacal microbiome alpha diversity and a shift in community structure between breeding stages, independent of treatment. While a previous study performed in male rufous‐collared sparrows (*Zonotrichia capensis*) found similar shifts in the microbiome between and across breeding seasons (Escallón et al., 2019), this is one of the few studies to find evidence for changes in the microbiome at a shorter timescale—within one breeding attempt—and in females (White et al., 2010). These changes in the cloacal bacterial community may be due to a variety of nonindependent, host‐specific factors that may change across breeding stages, including behavior, physiology, and immunity, as well as other factors, such as environment and diet. Overt behavioral changes occur in female birds across breeding stages as females invest time and energy on courtship solicitations and copulations during nest building and on incubation after egg‐laying (Norris & Lopez, 2011). Cloacal bacterial communities are more similar between niches that make contact more frequently (e.g., bird cloaca‐bird cloaca, bird cloaca‐nest; van Veelen et al., 2017). For example, pair‐bonded social partners experimentally inhibited from making cloacal contact during mating exhibited a decrease in overall cloacal microbiome diversity over time (White et al., 2010), suggesting that a shift from frequent copulations (e.g., during nest building) to less frequent copulations (e.g., during incubation) results in a decrease in cloacal microbiota. These behavioral changes are likely influenced by changes in hormone profiles across breeding stages, specifically with respect to temperate zone, seasonally breeding birds (Wingfield et al., 2000). For instance, circulating estradiol concentrations are consistently found to be highest when female birds are most sexually active (e.g., during nest building: Dawson, 1983; Norris & Lopez, 2011; Williams, 2012). Behavioral and physiological changes across breeding stages also likely influence immunological changes in the host (Milenkaya et al., 2013; Norris & Evans, 2000) that may indirectly affect the colonization and/or maintenance of cloacal bacteria. Finally, given that the cloaca is the terminus for the digestive tract in birds, any changes in diet across breeding stages may be associated with changes in the cloacal microbiome.

There are a number of possible reasons why there was not a short‐term effect of number of mates on cloacal microbiome diversity. First, we did not find a difference in the number of sires per brood when comparing estradiol‐ and blank‐implanted females, and this may be due to the consistent high rates of extra‐pair fertilizations in the population and/or limitations inherent in identifying the number of mates per individual. While we made the assumption that the number of sires per brood was a proxy for the number of mates per female, this assumption may not be valid for this tree swallow system since rates of extra‐pair activity are so high (almost 80%). As evidence of this, the proportion of sires per brood remained consistent (and high) between the females in the observational study and the subsequent experiment, even though we administered hormone implants to increase the sexual activity of the experimental females. The application of exogenous estradiol is an established method for increasing the frequency of solicitations and copulations in female birds (Moore, 1982). Nevertheless, across three breeding seasons, over 50% of sampled female tree swallows had broods sired by two distinct males, and 71%–78% of females had broods sired by two or three distinct males. There does not currently exist a method to comprehensively and accurately document the number of copulations secured by a free‐living bird. While spatial proximity data loggers, for example, may record how close two individuals are to each other, there is no way to deduce if the birds are mating, allopreening, fighting, or simply perched next to one another (Krause et al., 2013; Ryder et al., 2012). Although technological advances, such as the application of small video cameras in combination with proximity data loggers on free‐living birds, have helped contextualize social interactions (Rutz et al., 2007), interactions such as extra‐pair copulations can occur at night or just before dawn, when video capture is not possible.

In conclusion, we have shown how dynamic the cloacal microbiome of individuals can be both within and across breeding seasons in a free‐living bird species. We found support for a relationship between female age and the cloacal microbiome. Based on this age‐related pattern, we hypothesize that the effect of sexual activity on variation in the cloacal microbiome manifests over an individual's lifetime. An alternative hypothesis explaining age‐related changes in the microbiome may be that host‐associated microbiome diversity tracks changes in a host's changing investment in survival versus reproduction as they age. Further, the significant decrease in cloacal microbiome alpha diversity and the shift in overall community structure between nest building and incubation underscore the important role that the breeding stage plays in shaping the cloacal microbiome of a wild bird. Future studies should focus on longitudinally sampling the cloacal microbiome of individuals over the course of their entire life, with sampling occurring at multiple time points within a single breeding season, between breeding and nonbreeding stages (but see Escallón et al., 2019), and across breeding seasons.

## CONFLICT OF INTEREST

None declared.

## AUTHOR CONTRIBUTIONS

**Jessica Hernandez:** Conceptualization (lead); data curation (lead); formal analysis (lead); funding acquisition (lead); investigation (lead); methodology (lead); project administration (lead); supervision (lead); validation (lead); visualization (lead); writing–original draft (lead); writing–review and editing (lead). **Catherine Hucul:** Investigation (supporting); writing–review and editing (supporting). **Emily Reasor:** Investigation (supporting); writing–review and editing (supporting). **Taryn Smith:** Investigation (supporting); writing–review and editing (supporting). **Joel W. McGlothlin:** Formal analysis (supporting); methodology (supporting); resources (supporting); software (supporting); writing–review and editing (equal). **David C. Haak:** Formal analysis (supporting); methodology (supporting); validation (supporting); writing–review and editing (equal). **Lisa K. Belden:** Conceptualization (equal); data curation (equal); methodology (equal); resources (supporting); writing–review and editing (equal). **Ignacio T. Moore:** Conceptualization (equal); methodology (equal); resources (supporting); writing–review and editing (equal).

## Supporting information

Supplementary Material

## Data Availability

All data and related metadata files are available from the VTechData database (accession number https://doi.org/10.7294/P0HN‐AC48). The citation is "Hernandez, J. (2021). Tree swallow observational study and experiment dataset [Data set]. University Libraries, Virginia Tech. https://doi.org/10.7294/P0HN‐AC48".
