# Supplementary material for: Assessing age, breeding stage, and mating activity as drivers of variation in the reproductive microbiome of female tree swallows
Source: Ecol Evol. 2021 Jul 29;11(16):11398–413. doi: 10.1002/ece3.7929 (PMC8366841; doi:10.1002/ece3.7929)
Supplement: Supplementary file 1 — Supplementary Material [file ECE3-11-11398-s001.docx]

**Table S1. Characteristics of the eight microsatellite loci multiplexed in the observational study.** Primers for all eight microsatellite loci were previously cross-amplified in tree swallows (*Tachycineta bicolor*) in Makarewich et al. (2009). Loci were split into two PCR mixes (A or B) with respect to their annealing temperature. N = number of individuals genotyped. N_A_ = number of alleles. H_O_ = observed heterozygosity. H_E_ = expected heterozygosity.

| **Locus** | **Fluorescent**  **Label** | **Allele size range** | **Annealing temperature (°C)** | ***N*** | ***N*_A_** | ***H*_O_** | ***H*_E_** | **PCR**  **Mix** |
| --- | --- | --- | --- | --- | --- | --- | --- | --- |
| Tle19 | 6-FAM | 140-174 | 56 | 365 | 15 | 0.7918 | 0.8391 | A |
| Tle16 | PET | 227-249 | 56 | 365 | 10 | 0.6703 | 0.6767 | A |
| TaBi4 | VIC | 243-295 | 56 | 365 | 12 | 0.8959 | 0.8530 | A |
| TaBi8 | PET | 301-339 | 56 | 365 | 14 | 0.8324 | 0.8191 | A |
| TaBi1 | NED | 299-343 | 56 | 365 | 12 | 0.7041 | 0.7331 | A |
| TaBi25 | PET | 117-177 | 58 | 365 | 17 | 0.9836 | 0.7768 | B |
| TaBi34 | 6-FAM | 172-212 | 58 | 365 | 20 | 0.9699 | 0.8883 | B |
| Tal6 | VIC | 326-348 | 58 | 365 | 7 | 0.4740 | 0.6247 | B |

**Table S2. Characteristics of the ten microsatellite loci multiplexed in the experiment.** Primers for all ten microsatellite loci were previously cross-amplified in tree swallows (*Tachycineta bicolor*) in Makarewich et al. (2009). Loci were split into two PCR mixes (A or B) with respect to their annealing temperature. N = number of individuals genotyped. N_A_ = number of alleles. H_O_ = observed heterozygosity. H_E_ = expected heterozygosity.

| **Locus** | **Fluorescent**  **Label** | **Allele size range** | **Annealing temperature (°C)** | ***N*** | ***N*_A_** | ***H*_O_** | ***H*_E_** | **PCR**  **Mix** |
| --- | --- | --- | --- | --- | --- | --- | --- | --- |
| Tle19 | 6-FAM | 146-174 | 56 | 266 | 12 | 0.808 | 0.868 | A |
| Tle16 | PET | 227-251 | 56 | 266 | 8 | 0.707 | 0.802 | A |
| TaBi4 | VIC | 244-296 | 56 | 266 | 12 | 0.891 | 0.867 | A |
| TaBi8 | PET | 306-341 | 56 | 266 | 14 | 0.816 | 0.849 | A |
| TaBi1 | NED | 306-342 | 56 | 266 | 10 | 0.741 | 0.738 | A |
| TaBi25 | PET | 119-177 | 58 | 266 | 15 | 0.748 | 0.665 | B |
| TaBi34 | 6-FAM | 171-211 | 58 | 266 | 16 | 0.917 | 0.894 | B |
| Tal6 | VIC | 337-348 | 58 | 266 | 6 | 0.492 | 0.618 | B |
| Tal11 | NED | 195-215 | 58 | 266 | 10 | 0.650 | 0.627 | B |
| Tal8 | 6-FAM | 272-340 | 58 | 266 | 29 | 0.951 | 0.936 | B |

**Table S3. AICc-based model selection parameters and output for (A) amplicon sequence variants (ASV) Richness, (B) Shannon Index, and (C) Faith’s Phylogenetic Distance.** The reference age group is ‘SY’, which describes first-time breeding females.

(A) Model selection based on AICc for **ASV richness**.

| g1 = lm(Richness ~ Number of sires per brood *Female age) |
| --- |
| g2 = lm(Richness ~ Number of sires per brood +Female age) |
| g3 = lm(Richness ~ Female age) |
| g4 = lm(Richness ~ Number of sires per brood) |
| g5 = lm(Richness ~ 1) |

| Model | K | AICc | Delta_AICc | AICcWt | Cum.Wt | LL |
| --- | --- | --- | --- | --- | --- | --- |
| g3 | 3 | 796.82 | 0.00 | 0.76 | 0.76 | -395.23 |
| g5 | 2 | 799.85 | 3.03 | 0.17 | 0.92 | -397.84 |
| g2 | 6 | 801.92 | 5.10 | 0.06 | 0.98 | -394.31 |
| g4 | 5 | 804.81 | 7.99 | 0.01 | 1.00 | -396.94 |
| g1 | 9 | 808.81 | 11.98 | 0.00 | 1.00 | -393.93 |

(B) Model selection based on AICc for **Shannon Index**.

| g1 = lm(Shannon ~ Number of sires per brood *Female age) |
| --- |
| g2 = lm(Shannon ~ Number of sires per brood +Female age) |
| g3 = lm(Shannon ~ Female age) |
| g4 = lm(Shannon ~ Number of sires per brood) |
| g5 = lm(Shannon ~ 1) |

| Model | K | AICc | Delta_AICc | AICcWt | Cum.Wt | LL |
| --- | --- | --- | --- | --- | --- | --- |
| g3 | 3 | 240.76 | 0.00 | 0.72 | 0.72 | -117.20 |
| g5 | 2 | 243.24 | 2.48 | 0.21 | 0.92 | -119.53 |
| g2 | 6 | 245.88 | 5.12 | 0.06 | 0.98 | -116.28 |
| g4 | 5 | 248.08 | 7.31 | 0.02 | 1.00 | -118.58 |
| g1 | 9 | 252.21 | 11.44 | 0.00 | 1.00 | -115.63 |

(C) Model selection based on AICc for **Faith’s Phylogenetic Distance**.

| g1 = lm(Faith’s ~ Number of sires per brood*Female age) |
| --- |
| g2 = lm(Faith’s ~ Number of sires per brood +Female age) |
| g3 = lm(Faith’s ~ Female age) |
| g4 = lm(Faith’s ~ Number of sires per brood) |
| g5 = lm(Faith’s ~ 1) |

| Model | K | AICc | Delta_AICc | AICcWt | Cum.Wt | LL |
| --- | --- | --- | --- | --- | --- | --- |
| g3 | 3 | 454.53 | 0.00 | 0.71 | 0.71 | -224.08 |
| g5 | 2 | 456.88 | 2.35 | 0.22 | 0.93 | -226.35 |
| g2 | 6 | 459.79 | 5.26 | 0.05 | 0.98 | -223.24 |
| g4 | 5 | 461.92 | 7.39 | 0.02 | 1.00 | -225.50 |
| g1 | 9 | 467.11 | 12.58 | 0.00 | 1.00 | -223.08 |

**Table S4. Model summaries for the top ranked models from each model set in Table S3.** The response variables are bolded in the first column and the independent covariates specified in the top-ranking model(s) are in the second column. The reference age group is ‘SY’, which describes first-time breeding females. We include all top model summaries for which the delta AICc score is < 2.0 and below the null model. The ‘Intercept’ is included as a covariate if the null model was ranked highest.

| **Response** | **Model covariates** | **Estimate** | | **SE** | **t value** | ***P* value** |
| --- | --- | --- | --- | --- | --- | --- |
| ASV Richness | (Intercept) | 102.5 | 12.6 | | 8.1 | **<0.001** |
|  | Female Age | 36.2 | 15.8 | | 2.3 | **0.03** |
| Shannon | (Intercept) | 1.8 | 0.25 | | 7.3 | **<0.001** |
|  | Female Age | 0.68 | 0.32 | | 2.2 | **0.03** |
| Faith’s | (Intercept) | 17.5 | 1.1 | | 15.4 | **<0.001** |
|  | Female Age | 3.0 | 1.4 | | 2.1 | **0.04** |

**Table S5. AICc-based model selection parameters and output for (A) scaled-mass index and (B) hematocrit.** ‘SMi’ refers to scaled-mass index, a morphological condition metric (See Methods of Main Text). ‘Number of sires’ refers to the number of sires per brood, based on paternity analyses. ‘Female age’ refers to a female’s age, based on plumage scores.

Model selection based on AICc for **scaled-mass index**.

| g1 = lm(Scaled-mass index ~ Number of sires per brood *Female age) |
| --- |
| g2 = lm(Scaled-mass index ~ Number of sires per brood +Female age) |
| g3 = lm(Scaled-mass index ~ Female age) |
| g4 = lm(Scaled-mass index ~ Number of sires per brood) |
| g5 = lm(Scaled-mass index ~ 1) |

| Model | K | AICc | Delta_AICc | AICcWt | Cum.Wt | LL |
| --- | --- | --- | --- | --- | --- | --- |
| g4 | 4 | 252.56 | 0.00 | 0.58 | 0.58 | -121.91 |
| g2 | 5 | 254.92 | 2.36 | 0.18 | 0.76 | -121.90 |
| g5 | 2 | 255.22 | 2.66 | 0.15 | 0.92 | -125.50 |
| g3 | 3 | 257.33 | 4.77 | 0.05 | 0.97 | -125.45 |
| g1 | 7 | 258.65 | 6.09 | 0.03 | 1.00 | -121.23 |

Model selection based on AICc for **hematocrit**.

| g1 = lm(Hematocrit ~ Number of sires per brood *Female age) |
| --- |
| g2 = lm(Hematocrit ~ Number of sires per brood +Female age) |
| g3 = lm(Hematocrit ~ Female age) |
| g4 = lm(Hematocrit ~ Number of sires per brood) |
| g5 = lm(Hematocrit ~ 1) |

| Model | K | AICc | Delta_AICc | AICcWt | Cum.Wt | LL |
| --- | --- | --- | --- | --- | --- | --- |
| g4 | 4 | -230.43 | 0.00 | 0.36 | 0.36 | 119.61 |
| g5 | 2 | -230.34 | 0.09 | 0.35 | 0.71 | 117.28 |
| g3 | 3 | -228.58 | 1.84 | 0.14 | 0.86 | 117.52 |
| g2 | 5 | -228.37 | 2.06 | 0.13 | 0.99 | 119.78 |
| g1 | 7 | -223.71 | 6.72 | 0.01 | 1.00 | 120.02 |

**Table S6. Model summaries for the best performing model per body condition metric in Table S5.** The response variables are bolded in the first column and the independent covariates specified in the top-ranking model(s) are in the second column. The reference age group is ‘SY’, which describes first-time breeding females, and ‘Number of sires per brood 1’, which describes broods with one sire. We include all top model summaries for which the delta AICc score is < 2.0 and below the null model. The ‘Intercept’ is included as a covariate if the null model was ranked highest.

| **Response** | **Model covariates** | **Estimate** | **SE** | **t value** | ***P* value** |
| --- | --- | --- | --- | --- | --- |
| Scaled-mass index | (Intercept) | 20.2 | 0.57 | 35.6 | **<0.001** |
|  | Number of sires per brood 2 | 1.8 | 0.66 | 2.7 | **0.01** |
|  | Number of sires per brood 3+ | 1.5 | 0.80 | 1.9 | 0.06 |
| Hematocrit | (Intercept) | 0.51 | 0.01 | 57.2 | **<0.001** |
|  | Number of sires per brood 2 | 0.02 | 0.01 | 2.1 | **0.04** |
|  | Number of sires per brood 3+ | 0.01 | 0.01 | 1.1 | 0.29 |

**Table S7. Contrast summaries for body condition metrics in Table S6.**

| **Condition metric** | **Contrast** | **Estimate** | **SE** | **df** | **t ratio** | ***P* value** |
| --- | --- | --- | --- | --- | --- | --- |
| Scaled-mass index | 1 – 2 | -1.8 | 0.66 | 56 | -2.7 | **0.03** |
|  | 1 – (3+) | -1.5 | 0.80 | 56 | -1.9 | 0.15 |
|  | 2 – (3+) | 0.23 | 0.66 | 56 | 0.3 | 0.94 |
| Hematocrit | 1 – 2 | -0.02 | 0.01 | 53 | -2.1 | 0.10 |
|  | 1 – (3+) | -0.01 | 0.01 | 53 | -1.1 | 0.54 |
|  | 2 – (3+) | 0.01 | 0.01 | 53 | 0.9 | 0.63 |

**Table S8. Model summaries for the reproductive success (average brood mass, hatch success, fledging success) of females based on (A) age and (B) number of sires per brood.** The reference groups are ‘SY’, which describes first-time breeding females, and ‘Number of sires per brood 1’, which describes broods with one sire.

**(A) Reproductive success with respect to female age**

| **Reproductive success metric** | **Model covariates** | **Estimate** | **SE** | **t value** | **p value** |
| --- | --- | --- | --- | --- | --- |
| Average brood mass | (Intercept) | 9.0 | 1.2 | 7.4 | **<0.001** |
|  | Female age | 0.6 | 0.5 | 1.2 | 0.25 |
|  | Nestling age at sampling | 1.1 | 0.2 | 7.2 | **<0.001** |
| Fledging success | (Intercept) | 4.8 | 0.2 | 23 | **<0.001** |
|  | Female age | -0.5 | 0.3 | -1.9 | 0.07 |
| **Reproductive success metric** | **Model covariates** | **Estimate** | **SE** | **z value** | **p value** |
| Hatch success | (Intercept) | 2.2 | 0.1 | 21 | **<0.001** |
|  | Female age | -0.6 | 0.1 | -5.3 | **<0.001** |

**(B) Reproductive success with respect to number of sires per brood**

| **Reproductive success metric** | **Model covariates** | **Estimate** | **SE** | **t value** | **p value** |
| --- | --- | --- | --- | --- | --- |
| Average brood mass | (Intercept) | 8.7 | 1.3 | 6.5 | **<0.001** |
|  | Number of sires 2 | 1.3 | 0.7 | 1.7 | 0.09 |
|  | Number of sires 3+ | 1.2 | 0.9 | 1.3 | 0.18 |
|  | Nestling age at sampling | 1.0 | 0.2 | 6.0 | **<0.001** |
| Fledging success | (Intercept) | 4.2 | 0.3 | 15 | **<0.001** |
|  | Number of sires 2 | 0.5 | 0.3 | 1.6 | 0.12 |
|  | Number of sires 3+ | 0.8 | 0.4 | 1.9 | 0.06 |
| **Reproductive success metric** | **Model covariates** | **Estimate** | **SE** | **z value** | **p value** |
| Hatch success | (Intercept) | 1.9 | 0.1 | 14 | **<0.001** |
|  | Number of sires 2 | 0.1 | 0.1 | 1.0 | 0.32 |
|  | Number of sires 3+ | 0.3 | 0.2 | 1.6 | 0.12 |

**Table S9. AICc-based model selection parameters and output for circulating (log-transformed) 17*ß*-estradiol levels.** ‘Number of sires per brood’ refers to the number of sires per brood, based on paternity analyses. ‘Female age’ refers to a female’s age, based on plumage scores. The reference age group is ‘SY’, which describes first-time breeding females. Estradiol levels were natural log transformed.

| g1 =lm(Number of sires per brood ~ Log estradiol levels *Female age) |
| --- |
| g2 =lm(Number of sires per brood ~ Log estradiol levels +Female age) |
| g3 =lm(Number of sires per brood ~ Log estradiol levels) |
| g4 =lm(Number of sires per brood ~ Female age) |
| g5 =lm(Number of sires per brood ~ 1) |

| Model | K | AICc | Delta_AICc | AICcWt | Cum.Wt | LL |
| --- | --- | --- | --- | --- | --- | --- |
| g5 | 2 | 116.75 | 0.00 | 0.38 | 0.38 | -56.25 |
| g3 | 3 | 117.02 | 0.27 | 0.33 | 0.71 | -55.26 |
| g4 | 3 | 118.72 | 1.97 | 0.14 | 0.85 | -56.12 |
| g2 | 4 | 119.11 | 2.36 | 0.12 | 0.96 | -55.14 |
| g1 | 5 | 121.44 | 4.69 | 0.04 | 1.00 | -55.08 |

**Table S10. Model summaries for the top ranked models from each model set in Table S5.** The response variables are bolded in the first column and the independent covariates specified in the top-ranking model(s) are in the second column. The reference age group is ‘SY’, which describes first-time breeding females. We include all top model summaries for which the delta AICc score is < 2.0 and below the null model. The ‘Intercept’ is included as the only covariate if the null model was ranked highest.

| **Response** | **Model covariates** | **Estimate** | **SE** | **t value** | ***P* value** |
| --- | --- | --- | --- | --- | --- |
| Number of sires per brood | (Intercept) | 2.0 | 0.1 | 21 | **<0.001** |
|  | (Intercept) | 2.1 | 0.1 | 18 | **<0.001** |
|  | Log estradiol levels | -0.3 | 0.2 | -1.4 | 0.17 |
|  | (Intercept) | 2.1 | 0.2 | 13 | **<0.001** |
|  | Female age | -0.1 | 0.2 | -0.5 | 0.61 |

**Table S11. AICc-based model selection parameters and output for (A) amplicon sequence variant (ASV) richness, (B) Shannon index, and (C) Faith’s phylogenetic distance.**

(A) Model selection based on AICc for **ASV richness**.

| g1 = lm(ΔRichness ~ Treatment +Year, +Number of days implanted) |
| --- |
| g2 = lm(ΔRichness ~ Treatment +Year) |
| g3 = lm(ΔRichness ~ Treatment +Number of days implanted) |
| g4 = lm(ΔRichness ~ Year +Number of days implanted) |
| g5 = lm(ΔRichness ~ Treatment) |
| g6 = lm(ΔRichness ~ Year) |
| g7 = lm(ΔRichness ~ Number of days implanted) |
| g8 = lm(ΔRichness ~ 1) |

| Model | K | AICc | Delta_AICc | AICcWt | Cum.Wt | LL |
| --- | --- | --- | --- | --- | --- | --- |
| g7 | 3 | 474.66 | 0.00 | 0.42 | 0.42 | -234.03 |
| g4 | 4 | 475.17 | 0.51 | 0.33 | 0.75 | -233.07 |
| g6 | 3 | 477.65 | 2.98 | 0.09 | 0.84 | -235.52 |
| g8 | 2 | 478.01 | 3.34 | 0.08 | 0.92 | -236.86 |
| g3 | 5 | 479.52 | 4.86 | 0.04 | 0.96 | -233.97 |
| g1 | 6 | 480.37 | 5.70 | 0.02 | 0.98 | -233.05 |
| g2 | 5 | 482.37 | 7.70 | 0.01 | 0.99 | -235.39 |
| g5 | 4 | 482.47 | 7.80 | 0.01 | 1.00 | -236.72 |

(B) Model selection based on AICc for **the** **Shannon index**.

| g1 = lm(ΔShannon ~ Treatment +Year, +Number of days implanted) |
| --- |
| g2 = lm(ΔShannon ~ Treatment +Year) |
| g3 = lm(ΔShannon ~ Treatment +Number of days implanted) |
| g4 = lm(ΔShannon ~ Year +Number of days implanted) |
| g5 = lm(ΔShannon ~ Treatment) |
| g6 = lm(ΔShannon ~ Year) |
| g7 = lm(ΔShannon ~ Number of days implanted) |
| g8 = lm(ΔShannon ~ 1) |

| Model | K | AICc | Delta_AICc | AICcWt | Cum.Wt | LL |
| --- | --- | --- | --- | --- | --- | --- |
| g8 | 2 | 188.30 | 0.00 | 0.36 | 0.36 | -92.00 |
| g7 | 3 | 189.11 | 0.81 | 0.24 | 0.61 | -91.26 |
| g6 | 3 | 190.45 | 2.15 | 0.12 | 0.73 | -91.93 |
| g4 | 4 | 191.19 | 2.89 | 0.09 | 0.82 | -91.08 |
| g5 | 4 | 191.59 | 3.29 | 0.07 | 0.89 | -91.28 |
| g3 | 5 | 191.67 | 3.37 | 0.07 | 0.95 | -90.04 |
| g1 | 6 | 193.81 | 5.51 | 0.02 | 0.98 | -89.77 |
| g2 | 5 | 193.93 | 5.63 | 0.02 | 1.00 | -91.18 |

(C) Model selection based on AICc for **Faith’s phylogenetic distance**.

| g1 = lm(ΔFaith’s ~ Treatment +Year, +Number of days implanted, | data =data) |
| --- | --- |
| g2 = lm(ΔFaith’s ~ Treatment +Year, | data =data) |
| g3 = lm(ΔFaith’s ~ Treatment +Number of days implanted, | data =data) |
| g4 = lm(ΔFaith’s ~ Year +Number of days implanted, | data =data) |
| g5 = lm(ΔFaith’s ~ Treatment, | data =data) |
| g6 = lm(ΔFaith’s ~ Year, | data =data) |
| g7 = lm(ΔFaith’s ~ Number of days implanted, | data =data) |
| g8 = lm(ΔFaith’s ~ 1, | data =data) |

| Model | K | AICc | Delta_AICc | AICcWt | Cum.Wt | LL |
| --- | --- | --- | --- | --- | --- | --- |
| g4 | 4 | 299.70 | 0.00 | 0.52 | 0.52 | -145.34 |
| g6 | 3 | 301.81 | 2.11 | 0.18 | 0.70 | -147.61 |
| g7 | 3 | 301.85 | 2.15 | 0.18 | 0.88 | -147.63 |
| g1 | 6 | 304.80 | 5.09 | 0.04 | 0.92 | -145.26 |
| g8 | 2 | 304.99 | 5.28 | 0.04 | 0.96 | -150.35 |
| g2 | 5 | 306.03 | 6.33 | 0.02 | 0.98 | -147.23 |
| g3 | 5 | 306.67 | 6.96 | 0.02 | 1.00 | -147.54 |
| g5 | 4 | 309.03 | 9.32 | 0.00 | 1.00 | -150.00 |

**Table S12. Model summaries for the best performing model for (A) amplicon sequence variant (ASV) richness, (B) Shannon index, and (C) Faith’s phylogenetic distance in Table S10.** The response variables are bolded in the first column and the independent covariates specified in the top-ranking model are in the second column. We include all top model summaries for which the delta AICc score is < 2.0 and below the null model. The ‘Intercept’ is included as the only covariate if the null model was ranked highest. The reference sampling year is ‘2018’.

| **Response** | **Model covariates** | **Estimate** | | **SE** | **t value** | ***P* value** |
| --- | --- | --- | --- | --- | --- | --- |
| Change in ASV Richness | (Intercept) | -33.5 | 15.1 | | -2.2 | **0.03** |
|  | Days implanted | 1.4 | 0.58 | | 2.4 | **0.02** |
|  | (Intercept) | -11.9 | 21.9 | | -0.54 | 0.59 |
|  | Sampling year | -24.6 | 18.2 | | -1.4 | 0.18 |
|  | Days implanted | 1.3 | 0.58 | | 2.2 | **0.03** |
| **Response** | **Model covariates** | **Estimate** | **SE** | | **t value** | ***P* value** |
| Change in Shannon | (Intercept) | -0.05 | 0.30 | | -0.16 | 0.88 |
| **Response** | **Model covariates** | **Estimate** | **SE** | | **t value** | ***P* value** |
| Change in Faith’s PD | (Intercept) | -0.13 | 3.0 | | -0.04 | 0.97 |
|  | Sampling year | -5.3 | 2.5 | | -2.1 | **0.04** |
|  | Days implanted | 0.17 | 0.1 | | 2.1 | **0.04** |

**Table S13. PERMANOVA summaries for female cloacal bacterial community structure (based on Bray-Curtis and Jaccard,) during (A) nest building and (B) incubation.**

**(A) Nest building sampling period**

| **Distance metric** | **Model factors** | **df** | **F model** | **R^2^** | **Pr(>F)** |
| --- | --- | --- | --- | --- | --- |
| Bray-Curtis | Treatment | 2 | 1.0 | 0.05 | 0.39 |
|  | Residuals | 41 |  | 0.95 |  |
|  | Total | 43 |  | 1.0 |  |
| Jaccard | Treatment | 2 | 0.99 | 0.05 | 0.47 |
|  | Residuals | 41 |  | 0.95 |  |
|  | Total | 43 |  | 1.0 |  |

**(B) Incubation sampling period**

| **Distance metric** | **Model factors** | **df** | **F model** | **R^2^** | **Pr(>F)** |
| --- | --- | --- | --- | --- | --- |
| Bray-Curtis | Treatment | 2 | 1.5 | 0.07 | 0.06 |
|  | Residuals | 41 |  | 0.93 |  |
|  | Total | 43 |  | 1.0 |  |
| Jaccard | Treatment | 2 | 1.3 | 0.06 | 0.07 |
|  | Residuals | 41 |  | 0.94 |  |
|  | Total | 43 |  | 1.0 |  |

**Table S14. Betadisper summaries for female cloacal bacterial community structure (based on Bray-Curtis and Jaccard) during (A) nest building and (B) incubation.**

**(A) Nest building sampling period**

| **Distance metric** | **Model factors** | **df** | **F model** | **Pr(>F)** |
| --- | --- | --- | --- | --- |
| Bray-Curtis | Groups | 2 | 1.3 | 0.30 |
|  | Residuals | 41 |  |  |
| Jaccard | Groups | 2 | 1.7 | 0.19 |
|  | Residuals | 41 |  |  |

**(B) Incubation sampling period**

| **Distance metric** | **Model factors** | **df** | **F model** | **Pr(>F)** |
| --- | --- | --- | --- | --- |
| Bray-Curtis | Groups | 2 | 2.0 | 0.16 |
|  | Residuals | 41 |  |  |
| Jaccard | Groups | 2 | 2.3 | 0.11 |
|  | Residuals | 41 |  |  |

**Table S15. Model summaries for the reproductive success (average brood mass, hatch success, fledging success) of females per treatment group.** The reference group is ‘Treatment None’, which describes control females that were not implanted.

| **Reproductive success metric** | **Model covariates** | **Estimate** | **SE** | **t value** | **p value** |
| --- | --- | --- | --- | --- | --- |
| Average brood mass | (Intercept) | 6.1 | 1.4 | 4.4 | **<0.001** |
|  | Treatment Blank | -0.1 | 0.7 | -0.1 | 0.89 |
|  | Treatment Estradiol | 0.3 | 0.7 | 0.4 | 0.70 |
|  | Nestling age at sampling | 1.6 | 0.2 | 9.3 | **<0.001** |
| **Reproductive success metric** | **Model covariates** | **Estimate** | **SE** | **z value** | **p value** |
| Hatch success | (Intercept) | 0.9 | 0.2 | 5.5 | **<0.001** |
|  | Treatment Blank | -0.3 | 0.2 | -1.6 | 0.11 |
|  | Treatment Estradiol | -0.8 | 0.2 | -3.5 | **<0.001** |
| Fledging success | (Intercept) | 4.4 | 0.6 | 7.2 | **<0.001** |
|  | Treatment Blank | -0.7 | 0.8 | -0.9 | 0.35 |
|  | Treatment Estradiol | -1.6 | 0.8 | -2.1 | 0.05 |


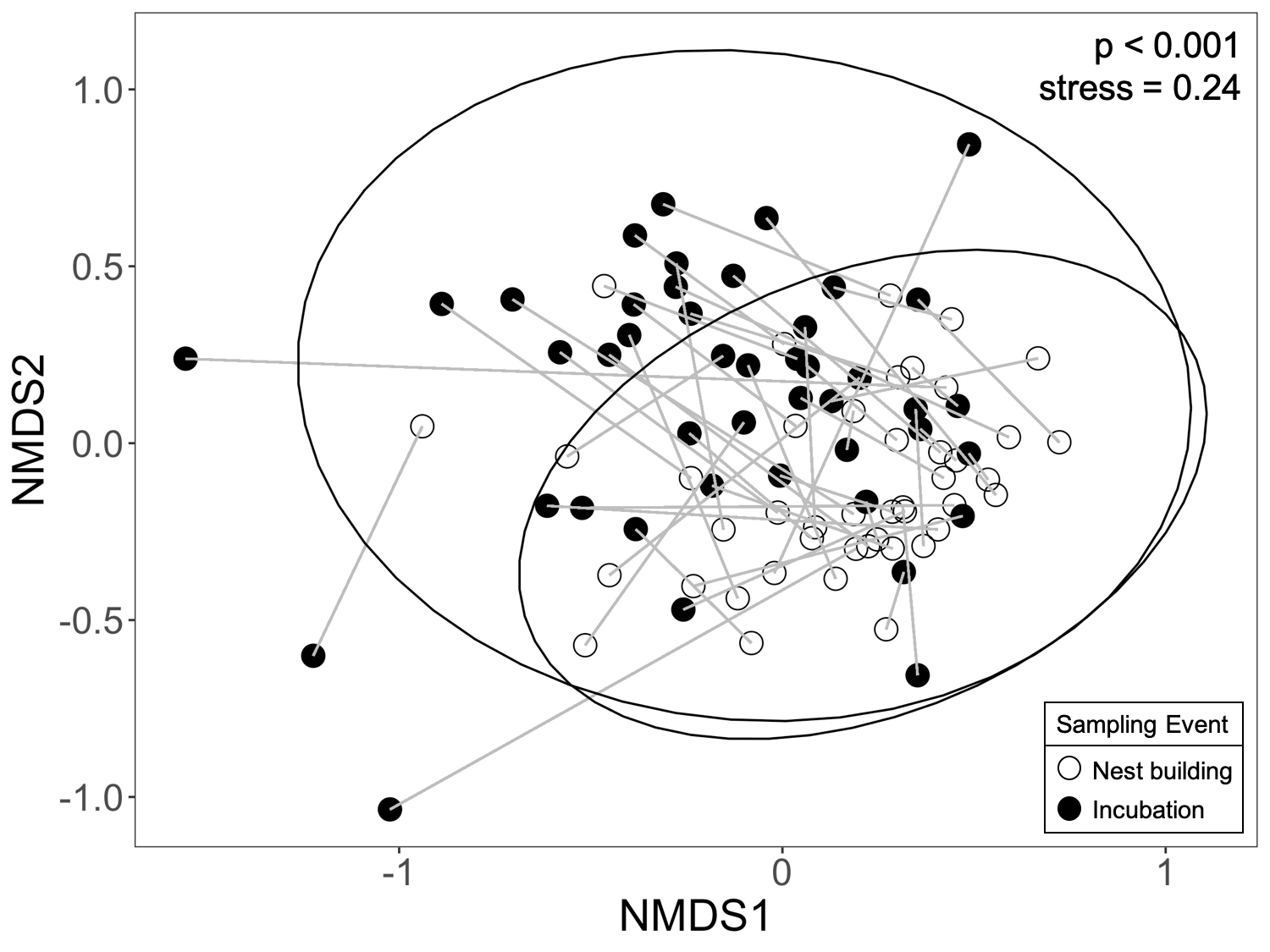


**Figure S1. Cloacal bacterial beta diversity (non-metric multidimensional scaling plot, NMDS based on Bray-Curtis dissimilarity) of female tree swallows at two breeding stages (nest building, incubation).** Each circle represents an individual female. Open circles = females sampled during nest building, filled circles = females sampled during incubation. Gray lines connect the two points per individual female. Circles closer together indicate individuals with more similar cloacal bacterial community composition. Sample sizes include *n* = 44 females per sampling period. NMDS based on Jaccard dissimilarity (not pictured here) is similar. P-value is based on a PERMANOVA model.
